# Flow cytometry-based quantification of genome editing efficiency in human cell lines using the *L1CAM* gene

**DOI:** 10.1371/journal.pone.0294146

**Published:** 2023-11-09

**Authors:** Muhammad Nazmul Hasan, Toshinori Hyodo, Mrityunjoy Biswas, Md. Lutfur Rahman, Yuko Mihara, Sivasundaram Karnan, Akinobu Ota, Shinobu Tsuzuki, Yoshitaka Hosokawa, Hiroyuki Konishi

**Affiliations:** 1 Department of Biochemistry, School of Medicine, Aichi Medical University, Nagakute, Aichi, Japan; 2 Department of Microbiology and Immunology, School of Medicine, Aichi Medical University, Nagakute, Aichi, Japan; Hirosaki University Graduate School of Medicine, JAPAN

## Abstract

CRISPR/Cas9 is a powerful genome editing system that has remarkably facilitated gene knockout and targeted knock-in. To accelerate the practical use of CRISPR/Cas9, however, it remains crucial to improve the efficiency, precision, and specificity of genome editing, particularly targeted knock-in, achieved with this system. To improve genome editing efficiency, researchers should first have a molecular assay that allows sensitive monitoring of genome editing events with simple procedures. In the current study, we demonstrate that genome editing events occurring in *L1CAM*, an X-chromosome gene encoding a cell surface protein, can be readily monitored using flow cytometry (FCM) in multiple human cell lines including neuroblastoma cell lines. The abrogation of *L1CAM* was efficiently achieved using Cas9 nucleases which disrupt exons encoding the L1CAM extracellular domain, and was easily detected by FCM using anti-L1CAM antibodies. Notably, *L1CAM*-abrogated cells could be quantified by FCM in four days after transfection with a Cas9 nuclease, which is much faster than an established assay based on the *PIGA* gene. In addition, the *L1CAM*-based assay allowed us to measure the efficiency of targeted knock-in (correction of *L1CAM* mutations) accomplished through different strategies, including a Cas9 nuclease-mediated method, tandem paired nicking, and prime editing. Our *L1CAM*-based assay using FCM enables rapid and sensitive quantification of genome editing efficiencies and will thereby help researchers improve genome editing technologies.

## Introduction

The CRISPR/Cas system is an advanced genome editing technology that offers increased efficiency and remarkable convenience for genome engineering [1]. Gene knockout and targeted knock-in are achieved through the repair of double-strand breaks (DSBs) created by wild-type CRISPR/Cas (Cas nuclease) at predetermined genomic positions [2, 3]. In mammalian cells, knockout is readily accomplished because DSBs are efficiently processed via error-prone non-homologous end-joining, which results in frequent insertions and deletions (indels) and shifts in reading frames. In contrast, DSB-based targeted knock-in has insufficient precision and specificity because of frequent indels occurring at on-target and off-target genomic loci [3, 4]. To overcome this issue, various Cas9 nickase-based methods for precise targeted knock-in have been developed. Such methods include those introducing nicks into both genomic DNA (gDNA) and donor DNA to increase the efficiency of targeted knock-in [5–7], base editing mediated by deaminases fused with Cas9 nickases to create programmed single-base substitutions [8–10], and prime editing mediated by a reverse transcriptase (RT) fused with Cas9 nickases to promote different types of precision genome editing [11]. Nonetheless, to enable future practical use of these methods in a broad range of applications, it remains crucial to improve and optimize these methods to ensure high efficiency, precision, and specificity of targeted knock-in in various experimental settings.

To technically improve targeted knock-in, it is necessary, as a prerequisite, to have a molecular system that sensitively monitors the incidence of targeted knock-in using simple procedures. T7E1 and Surveyor assays are commonly used to determine the ratios of edited versus intact DNA strands [2, 12], but they cannot distinguish correctly edited DNA strands from unwanted indels. Although many previous studies have performed restriction fragment length analysis to quantify the efficiency of targeted knock-in, this method has the drawback of insufficient sensitivity. Another common strategy is deep sequencing analysis of edited genomic loci using high-throughput sequencing (HTS) [3]. However, HTS remains costly for routine use with a large series of samples.

In an attempt to circumvent the above-mentioned issues, we developed and utilized a molecular system that reports the efficiency of targeted knock-in harnessing the *PIGA* gene as a platform. *PIGA*, located on the X-chromosome, is required for the biosynthesis of glycosylphosphatidylinositol (GPI)-anchors which protrude outward from the cell membrane [13, 14]. Once a *PIGA* allele is disrupted in male diploid cells, GPI-anchors are lost from the cell surface, which is readily detectable through FCM [15, 16]. We then isolated a near-diploid male cell clone in which the *PIGA* allele was inactivated by the insertion of an ectopic stop codon. Correction of the *PIGA* mutation in this clone by targeted knock-in of the wild-type *PIGA* sequence leads to the recovery of GPI-anchors on the cell surface, and FCM analysis of the resultant cells reports the percentage of *PIGA*-corrected cells which represents the efficiency of targeted knock-in [7]. This assay (designated as *PIGA* correction assay) has proven helpful in evaluating the efficiency of targeted knock-in because of its sensitivity, substantially low background, high-throughput nature, and simple and cost-effective procedures.

In addition, distinct molecular systems that monitor the efficiency of targeted knock-in via FCM have been generated by exploiting the *PIGP* and *CD55* genes [17, 18]. *PIGP* is another gene essential for producing GPI-anchors, and CD55 is a GPI-anchored protein distributed over the cell membrane. The activity and inactivity of these genes can therefore be evaluated by analyzing cells using FCM, enabling sensitive monitoring of the efficiency of targeted knock-in similar to the *PIGA* correction assay. However, because both *PIGP* and *CD55* are autosomal, researchers must first inactivate one allele of these reporter genes to accurately measure the frequency of reporter gene editing. To evaluate targeted knock-in in *PIGP* and *CD55*, an allele of these reporter genes should be eliminated and the other mutated, to enable the wild-type donor DNA to undergo homology-directed recombination only with a reporter allele. Such genome manipulation requires significant time and effort to quantify the incidence of targeted knock-in. Furthermore, the above-mentioned assays, based on *PIGA*, *PIGP*, and *CD55*, rely on the GPI-anchor biosynthesis pathway. Accordingly, these assays do not function when GPI-anchor synthesis is impaired because of alterations or loss of expression in one of the many GPI-anchor biosynthesis genes.

We therefore sought to develop another molecular system that allows FCM-based sensitive quantification of targeted knock-in events that harnesses an X-chromosomal gene not involved in GPI-anchor biosynthesis. The *L1CAM* gene, located on the X-chromosome, produces a protein protruding from cell membranes [19, 20] and exhibits robust expression in many human cell lines, according to the Expression Atlas database (https://www.ebi.ac.uk/gxa/home). Consequently, in this study, we investigated whether *L1CAM* can be exploited as a reporter to monitor the efficiency of targeted knock-in using an FCM-based assay. In addition, the utility of the FCM-based assay in detecting *L1CAM* gene disruption was also assessed.

## Materials and methods

### General molecular biology procedures

gDNA extraction from SK-N-BE(2) cells was performed using a PureLink Genomic DNA Mini Kit (Thermo Fisher Scientific, Waltham, MA, USA) following the manufacturer’s protocol. For complementary DNA (cDNA) synthesis, total RNA was isolated from SK-N-BE(2) cells using a Nucleospin RNA (Macherey-Nagel, Düren, Germany) and converted to cDNA using a High Capacity cDNA Reverse Transcription Kit (Thermo Fisher Scientific) according to the manufacturers’ instructions. PCR was performed using KAPA HiFi HotStart ReadyMix (Roche, Basel, Switzerland) to prepare the reaction mixture and a MiniAmp Plus thermal cycler (Thermo Fisher Scientific) for amplification. AE-6932GXES Printgraph (ATTO, Tokyo, Japan) was used to image PCR products and other DNA fragments electrophoresed on agarose gels. DNA fragments were recovered from agarose gels using a Gel/PCR DNA Isolation System (Viogene, New Taipei, Taiwan). Sanger sequencing was carried out using a BigDye Terminator v3.1 Cycle Sequencing Kit (Thermo Fisher Scientific) to prepare the reaction mixtures, a MiniAmp Plus thermal cycler for reaction, and a 3500 genetic analyzer (Thermo Fisher Scientific) for electrophoretic analysis.

### Plasmids

pSpCas9(BB)-2A-Puro (PX459) V2.0 (Addgene #62988) and pSpCas9n(BB)-2A-Puro (PX462) V2.0 (#62987) were obtained as generous gifts from Dr. Feng Zhang (Broad Institute), while pCMV-PE2-P2A-GFP (#132776), pCMV-PEmax-P2A-GFP (#180020), pU6-pegRNA-GG-acceptor (#132777), pU6-tmpknot-GG-acceptor (#174039), and pU6-tevopreq1-GG-acceptor (#174038) were generous gifts from Dr. David Liu (Broad Institute). Plasmids expressing Cas9 nucleases, Cas9 (D10A) nickases, and Cas9 (H840A) nickases were constructed based on PX459, PX462, and pH840Apuro [7], respectively, following Feng Zhang laboratory’s protocol [21]. The target sequences of individual Cas9 nucleases and nickases are listed in S1 Table. Potential off-target sites for respective Cas9 nucleases and nickases were deduced using the CRISPOR website (http://crispor.tefor.net/) [22] and are listed in S2 Table. In assays where these Cas9 plasmids were transfected into cells, PX459, PX462 and pH840Apuro were used as vector controls for Cas9 nucleases, D10A nickases, and H840A nickases, respectively.

To construct prime editing guide RNA (pegRNA) plasmids, synthetic transcription cassettes expressing pegRNAs, engineered pegRNAs (epegRNAs), and non-target controls (S1 Fig) were created by annealing six 5’-phophorylated oligonucleotides together and incorporating the resultant DNA assembly into one of three versatile vectors (pU6-pegRNA-GG-acceptor, pU6-tmpknot-GG-acceptor, and pU6-tevopreq1-GG-acceptor) at the BsaI-BsaI site.

To create Donor-*L1CAM*, a 1.9-kb DNA fragment comprising the wild-type *L1CAM* genomic sequence was PCR-amplified using a forward primer located on intron 11 and a reverse primer located on exon 17 (S3 Table), and incorporated into the XbaI-XhoI site of pBluescript II KS (+) (Agilent Technologies, Santa Clara, CA, USA). PCR-amplified portions within the constructed plasmids were verified by Sanger sequencing. The entire sequence of Donor-*L1CAM* is shown in S2 Fig.

### Cell culture

SK-N-BE(2) cells (CRL-2271, American Type Culture Collection (ATCC), Manassas, VA, USA) were cultured in a 1:1 mixture of E-MEM (Fujifilm, Tokyo, Japan) and Ham’s F-12 (Fujifilm) supplemented with 10% fetal bovine serum (FBS; Merck, Darmstadt, Germany) and 1% penicillin and streptomycin (P&S; Fujifilm). CHP-134 and LA-N-5 cells (RCB0487 and RCB0485, respectively, Riken BioResource Research Center, Tsukuba, Japan) were cultured in RPMI-1640 (Fujifilm) supplemented with 10% FBS and 1% P&S; TGW cells (JCRB0618, Japanese Collection of Research Bioresources Cell Bank, Ibaraki, Japan) were cultured in E-MEM supplemented with 10% FBS and 1% P&S; and HCT116 cells (CCL-247, ATCC) were cultured in McCoy’s 5A (modified) medium (Thermo Fisher Scientific) supplemented with 5% FBS and 1% P&S. SK-N-BE(2)-derived *L1CAM* knockout cells and reporter cell clones were cultured under the same conditions as their parental cell line. Cells were cultured in a humidified incubator MCO-170AICUVD-PJ (PHCbi, Tokyo, Japan) with 5% CO_2_ at 37°C. To transfect SK-N-BE(2), LA-N-5, TGW, HCT116, and derivative cell clones, 1 × 10^6^ cells were electroporated with plasmids (1 μg each, unless otherwise noted) using a 4D-Nucleofector system (Lonza, Basel, Switzerland) according to the manufacturer’s instructions. To transfect CHP-134 cells, FuGENE6 Transfection Reagent (Promega, Madison, WI, USA) was used as per the manufacturer’s instructions.

### Immunocytochemistry, FCM analysis, and cell sorting

The following antibodies were diluted to the indicated concentrations with phosphate-buffered saline containing 0.25 mM EDTA and 0.4% FBS, and used to stain cultured cells: anti-human L1CAM mouse monoclonal antibody 5G3 (2.5 μg/mL; sc-33686, Santa Cruz Biotechnology, Dallas, TX, USA; Antibody Registry AB_626934), anti-human L1CAM mouse monoclonal antibody UJ127.11 (2.5 μg/mL; sc-53386, Santa Cruz Biotechnology; Antibody Registry AB_628937), phycoerythrin (PE)-conjugated anti-human L1CAM mouse monoclonal antibody 5G3 (200-fold dilution; 564193, BD Biosciences, Franklin Lakes, NJ, USA; Antibody Registry AB_2738660), Alexa Fluor 488-conjugated anti-mouse IgG (H+L) goat secondary antibody (0.02 μg/mL; A32723, Thermo Fisher Scientific; Antibody Registry AB_2633275), and Alexa Fluor 594-conjugated anti-mouse IgG (H+L) goat secondary antibody (0.02 μg/mL; ab150116, Abcam, Cambridge, U.K.; Antibody Registry AB_2650601).

To stain the L1CAM protein, cells were detached using Accutase (Innovative Cell Technologies, San Diego, CA, USA) and incubated with primary and secondary antibodies for 60 and 20 min, respectively, under light protective conditions. The cells were then analyzed using an LSRFortessa X-20 Flow Cytometer (BD Biosciences). FACSAria III (BD Biosciences) was used for sorting and clonal isolation of L1CAM-positive and -negative cells. To stain the GPI-anchors distributed on the cell membrane, cells were treated with 0.1 μg/ml fluorescence-labeled aerolysin (FLAER; Cedarlane, Ontario, Canada) dissolved in phosphate-buffered saline containing 0.25 mM EDTA and 0.4% FBS.

### Creation of *L1CAM*-mutant reporter clones

Initially, two donor plasmids carrying the same stretch of the *L1CAM* genomic region as Donor-*L1CAM* but containing mutations in *L1CAM* exon 14 (mut-1 and mut-2, respectively) were created by replacing the relevant portions of the *L1CAM* genomic sequence in Donor-*L1CAM* with PCR-amplified mutant DNA fragments. Subsequently, SK-N-BE(2) cells were transfected with the constructed *L1CAM*-mutant donor plasmids along with a pair of Cas9 (D10A) nickases, #2 and #6, to create two reporter clones harboring different mutations within *L1CAM* exon 14 via tandem paired nicking (TPN)-based targeted knock-in. Four days later, the transfected cells were processed for fluorescent labeling of the L1CAM protein, FCM analysis, and the collection of L1CAM-negative cells using a cell sorter. Two L1CAM-negative cell clones, harboring *L1CAM* mut-1 and mut-2 respectively, were established, and the sequences of incorporated mutations along with the entire 1,922-bp *L1CAM* genomic sequence corresponding to the donor DNA fragments were verified. Proliferation indices in the isolated cell clones and parental SK-N-BE(2) cells were compared to address whether immunostaining followed by cell sorting had altered the growth properties of the cells, and no appreciable differences in proliferation indices among these cells were exhibited (S3 Fig).

### 3-(4,5-Dimethylthiazol-2-yl)-2,5-Diphenyltetrazolium Bromide (MTT) assay

For the MTT assay, 1.0 × 10^3^ cells were seeded into each well of five 96-well tissue culture plates with 100 μL/well medium. At 24, 48, 72, 96 and 120 h after seeding, cells in one 96-well plate were supplemented with 10 μL/well (final concentration 0.5 mg/mL) of the MTT labeling reagent, a component of Cell Proliferation Kit I (Roche), and incubated for 5 h. Cells were then treated overnight with 100 μL/well solubilization buffer included in the kit, and absorbance at 595 nm was measured using a SpectraMax M5 Microplate Reader (Molecular Devices, San Jose, CA, USA).

### Correction of *L1CAM*-inactivating mutations

Correction of *L1CAM*-inactivating mutations (mut-1 and mut-2) via Cas9 nuclease-based and TPN-based targeted knock-in strategies was performed by transfecting the *L1CAM*-mutant reporter clones with a plasmid expressing a Cas9 nuclease or a pair of plasmids expressing Cas9 nickases, along with Donor-*L1CAM*. After three days of incubation, the transfected cells were treated with anti-L1CAM monoclonal antibody 5G3 and Alexa Flour 488-conjugated secondary antibody. The cells were then FCM-analyzed to determine the ratios of *L1CAM*-positive cells.

To conduct prime editing, reporter clones were transfected with pCMV-PE2-P2A-GFP or pCMV-PEmax-P2A-GFP along with one of the plasmids expressing pegRNAs, epegRNAs (“tmpknot”, a trimmed pseudoknot from Moloney murine leukemia virus or “tevopreQ_1_”, a modified and trimmed prequeosine1-1 riboswitch aptamer), or their respective controls (S1 Fig). Four days later, the transfected cells were stained using PE-conjugated anti-human L1CAM mouse monoclonal antibody 5G3, and then processed for FCM to detect *L1CAM*-positive cells by drawing FL1-A (586 ± 7.5 nm)/FL2-A (450 ± 25 nm) dot plots under 561-nm/405-nm laser excitation.

### *L1CAM* sequencing in FCM-sorted cells

Cells were initially transfected with a Cas9 nuclease, or Cas9 nickases along with Donor-*L1CAM*, to edit *L1CAM*, and were processed for FCM-based sorting in which Alexa Fluor 488-positive and -negative cell populations were isolated. gDNA was extracted from the isolated cells and used as templates for PCR amplification of genomic regions encompassing the *L1CAM* intron 25–exon 26 boundary (for knockout by Cas9 nuclease) or exon 14 (targeted knock-in via TPN). The resulting bulk PCR products were digested with EcoRI and BamHI and ligated to the EcoRI–BamHI site of pBluescript II KS (+). Competent cells were then transformed with ligation mixtures, spread onto Lysogeny Broth (LB) agar plates containing ampicillin, and incubated overnight. Multiple bacterial clones formed on the LB plates were selected, cultured in LB liquid medium with ampicillin, and subjected to plasmid preparation. Lastly, the PCR products incorporated within the plasmids were Sanger sequenced. The primers used for PCR amplification and Sanger sequencing are listed in S3 Table.

### Statistical analysis

All statistical analyses were conducted using Intercooled Stata (StataCorp, College Station, TX, USA). Methods employed for statistical analyses on the respective experimental data and the outcomes of the analyses are documented in the corresponding figure legends. When three or five independent experiments were performed for statistical analysis, no replicate samples were included in each experiment unless otherwise noted.

## Results

### *L1CAM* disruption in SK-N-BE(2) cells can be quantified using FCM

**Fig 1:**
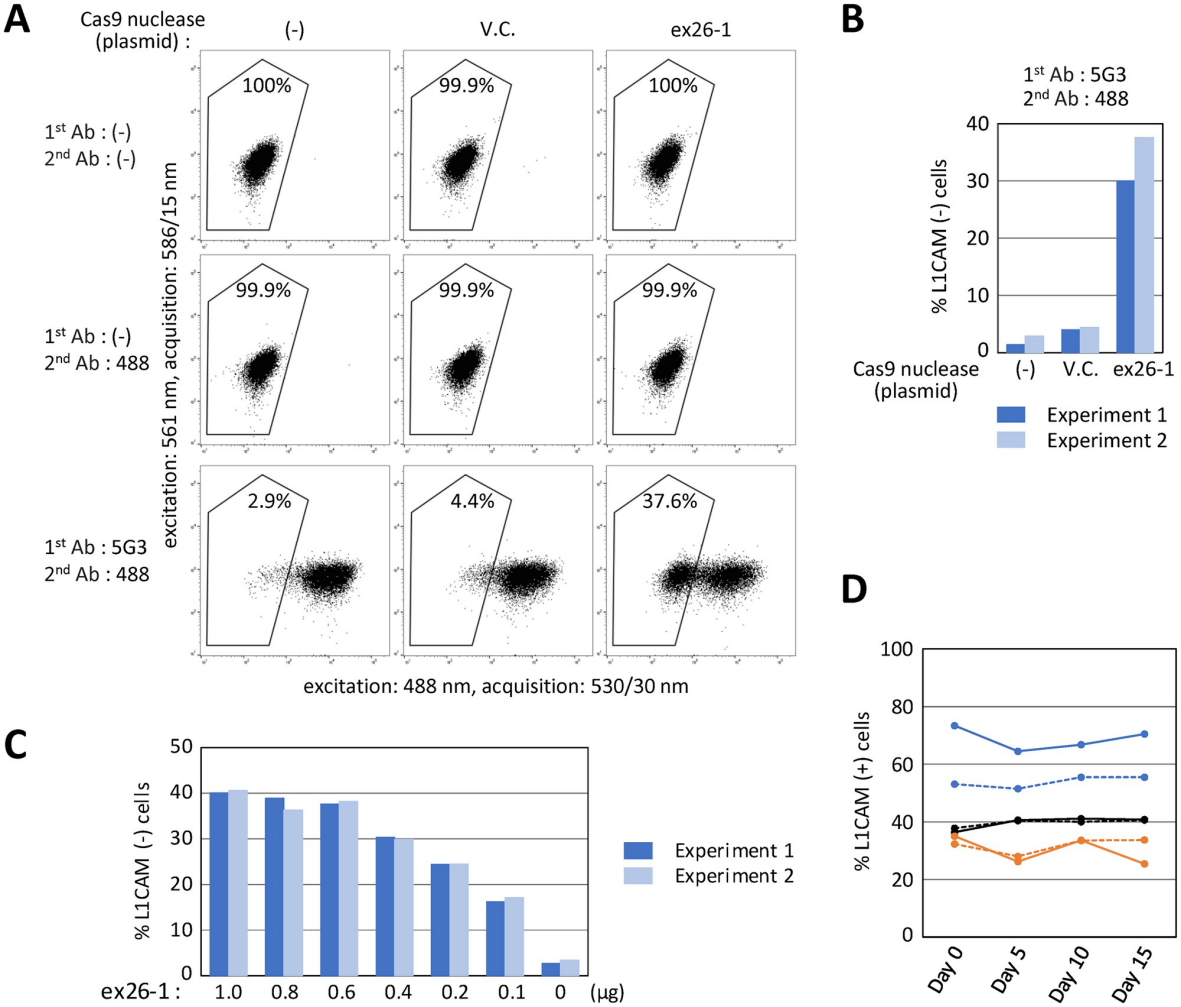
*L1CAM* disruption can be quantified using FCM. **(A)** Representative dot plots obtained from FCM analyses. SK-N-BE(2) cells were transfected with a plasmid listed at the top, stained with primary and secondary antibodies shown on the left, and FCM-analyzed. The percentages of Alexa Fluor 488-negative cells are denoted in the plots. In the figures shown hereafter, abbreviations are used as follows: ex26-1, a Cas9 nuclease targeted to the boundary of *L1CAM* intron 25 and exon 26; V.C., vector control; 5G3, anti-L1CAM monoclonal antibody 5G3; 488, Alexa Fluor 488-conjugated secondary antibody. **(B)** Graphical summary of experimental results, with representatives shown in (A). Only the results obtained using 5G3 and 488 antibodies are summarized. **(C)** SK-N-BE(2) cells were transfected with the indicated amounts of ex26-1 nuclease plasmid and analyzed similarly to (A) and (B). **(D)** The ratios of L1CAM-positive and -negative SK-N-BE(2) cells within the cell mixture over a 15-day incubation period. SK-N-BE(2) cells comprising L1CAM-positive and -negative populations at six different intermixing ratios (shown by six polygonal lines) were analyzed using FCM at four sequential time points (indicated at the bottom) during a 15-day incubation period. No significant differences in the percentages of L1CAM-positive cells were found among data obtained at four different time points (one-way repeated measures ANOVA).

We initially addressed whether *L1CAM* disruption using Cas9 nuclease could be detected through fluorescent labeling of its protein product distributed over the cell surface followed by FCM analysis. A near-diploid neuroblastoma cell line derived from a male infant, SK-N-BE(2), was transfected with a Cas9 nuclease disrupting the boundary of *L1CAM* intron 25 and exon 26 (ex26-1), and stained using an anti-L1CAM monoclonal antibody (5G3) and Alexa Fluor 488-conjugated secondary antibody at 4 days after transfection. We found that *L1CAM* disruption resulted in the emergence of an Alexa Fluor 488-negative cell population upon FCM in this experimental setting (Fig 1A and 1B). The L1CAM-negative cell population was reduced when the cells were transfected with lower amounts of ex26-1 nuclease (Fig 1C). The Alexa Fluor 488-positive and -negative cell populations were then isolated via FCM-based sorting and subjected to *L1CAM* genotyping. This analysis verified that negativity but not positivity for Alexa Fluor 488 represents the *L1CAM* disruption by indels (S4 Fig). In addition, we found that *L1CAM*-disrupted SK-N-BE(2) cells grow at a similar rate as *L1CAM*-intact SK-N-BE(2) cells for 15 days (Fig 1D). These data indicate that FCM-based quantification of *L1CAM*-disrupted SK-N-BE(2) cells is feasible using anti-L1CAM antibody (5G3). This FCM-based assay is hereafter referred to as “*L1CAM* assay”.

### Disruption of the L1CAM extracellular domain allows the detection of L1CAM-negative cells by FCM

**Fig 2:**
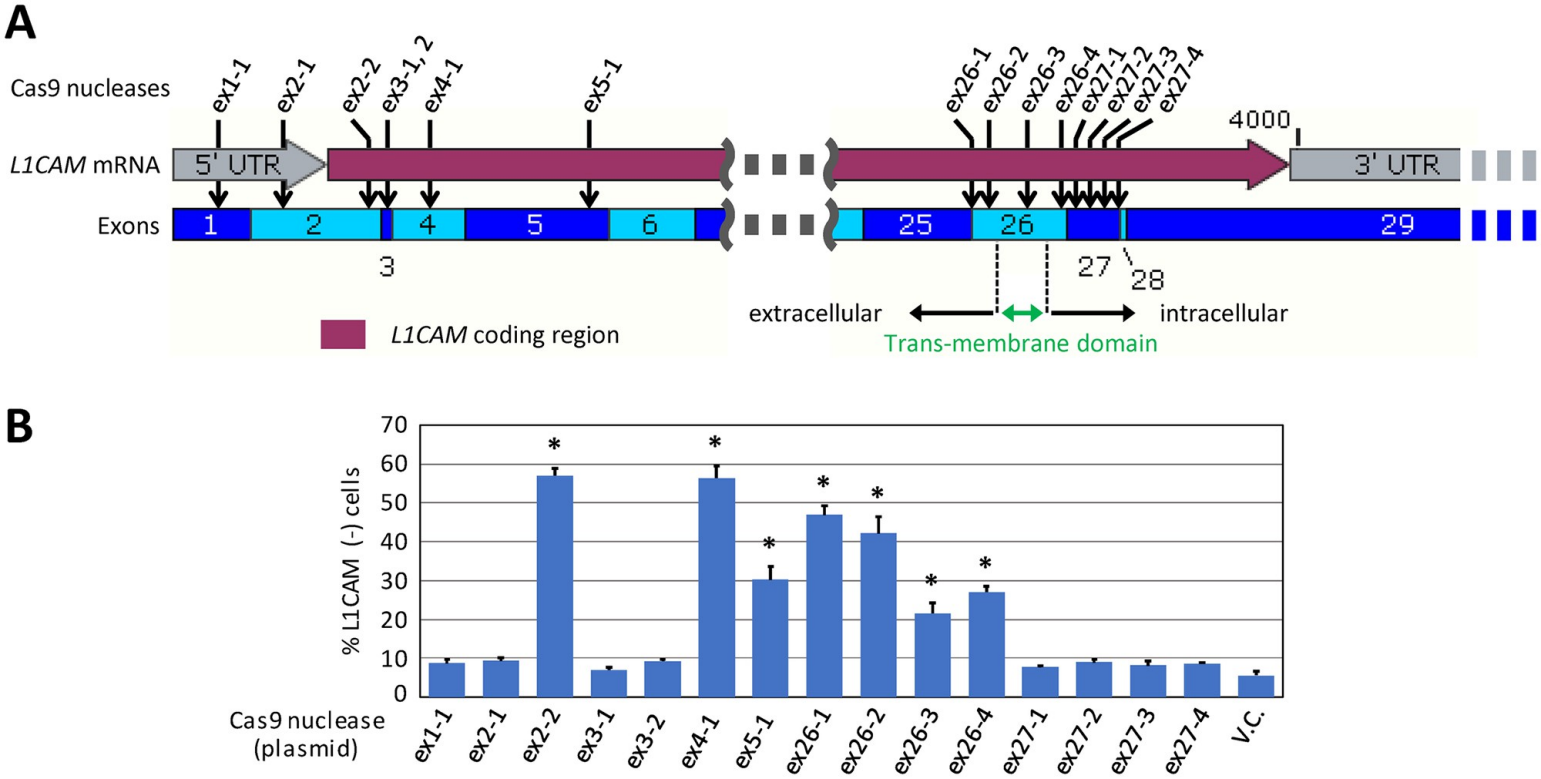
Disruption of the L1CAM extracellular domain allows the detection of L1CAM-negative cells by FCM. **(A)** Schematic map of *L1CAM* cDNA (NM_001278116.2). Down arrows indicate the target sites disrupted by Cas9 nucleases. Numbers noted in dark and light blue boxes indicate exons. **(B)** SK-N-BE(2) cells were transfected with the indicated Cas9 nuclease plasmids and analyzed similarly to Fig 1A–1C. Data represent the mean and standard error of the mean (SEM) values from five independent experiments. * *p* < 0.001 compared with V.C., one-way analysis of variance (ANOVA) with post-hoc Bonferroni test.

*L1CAM* has a total of 29 exons (based on NM_001278116.2). Its coding sequence starts in exon 2, ends in the last exon, and comprises a region coding a trans-membrane domain within exon 26. We investigated which *L1CAM* exons should be disrupted to effectively generate L1CAM-negative cells for FCM-based monitoring. To address this, we transfected SK-N-BE(2) cells with one of 15 Cas9 nucleases targeted to exons 1–5, 26, and 27 (Fig 2A), and assessed the efficiency of *L1CAM* disruption by analyzing the transfectants using FCM with the same procedures as mentioned above. It was found that efficient *L1CAM* disruption was achieved by transfecting cells with Cas9 nucleases targeted to exons coding the L1CAM extracellular domain (ex2-2, ex4-1, ex5-1, ex26-1, and ex26-2; Fig 2B). This is likely because Cas9-based disruption of the extracellular domain causes indels at the disrupted site in the genome, leading to a shift in the *L1CAM* reading frame and the abrogation of its trans-membrane domain. As an exception, Cas9 nucleases targeted to exon 3 (ex3-1 and ex3-2) did not increase Alexa Fluor 488-negative cells. This is probably because *L1CAM* exon 3, consisting of 15 nucleotides, is an alternative exon as suggested by its absence in another form of *L1CAM* mRNA (NM_001143963.2). Indeed, our RT-PCR analysis across *L1CAM* exons 2–4 amplified two intermixed PCR products of different sizes, and the sequencing of these products indicated the absence of exon 3 within a subset of *L1CAM* mRNA, supporting the alternative splicing of exon 3 (S5 Fig).

Cas9 nucleases whose target sequences reside within or downstream of the trans-membrane domain (ex26-3, ex26-4, and ex27-1–4) yielded fewer Alexa Fluor 488-negative cells, probably because an incomplete abrogation of the L1CAM trans-membrane domain allowed the extracellular domain to remain on the cell membrane in a subset of transfectants. Such cells should remain Alexa Fluor 488-positive because the 5G3 antibody recognizes the extracellular portion of L1CAM [23]. No significant increase in Alexa Fluor 488-negative cells was detected when Cas9 nucleases were targeted to genomic portions relatively far downstream of the trans-membrane domain (ex27-1–4) or within the 5′ untranslated region of the *L1CAM* gene (ex1-1 and ex2-1). These data collectively suggest that exons coding the L1CAM extracellular domain should be targeted for disruption to effectively monitor *L1CAM* inactivation events by FCM.

### *L1CAM*-disrupted SK-N-BE(2) cells can be quantified by FCM four days after Cas9 transfection

**Fig 3:**
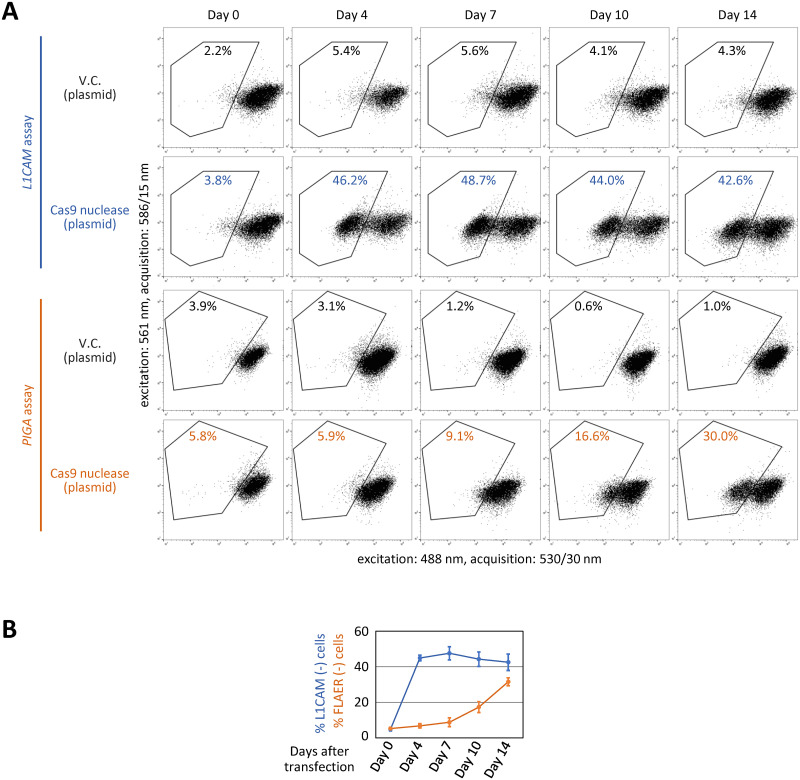
*L1CAM* disruption can be detected after a shorter cell incubation time than *PIGA* disruption. **(A)** Representative dot plots obtained from FCM analyses. SK-N-BE(2) cells were transfected with a plasmid shown on the left, and stained and FCM-analyzed after the incubation periods indicated at the top. Cas9 nuclease plasmids ex26-1 and *PIGA*-D [7] were used to disrupt *L1CAM* and *PIGA*, respectively. Anti-L1CAM antibody 5G3 and Alexa Fluor 488-conjugated secondary antibody were used to detect *L1CAM* disruption, while fluorescence-labeled aerolysin (FLAER) [24, 25] was used to detect *PIGA* disruption. The percentages of fluorescence-negative cells are denoted in the plots. **(B)** A graphical summary of the experimental results, with representatives shown in (A). The results of cell transfection with vector control plasmids were omitted from the graph. Data represent the mean and SEM values from three independent experiments.

We next sought to determine the optimal cell incubation time from Cas9 nuclease-based *L1CAM* disruption to the *L1CAM* assay. We initially transfected SK-N-BE(2) cells with a Cas9 nuclease against *L1CAM* (ex26-1) and, at 4, 7, 10, and 14 days after transfection, stained the cells and quantified the *L1CAM*-disrupted cells using FCM as described above. As a result, the ratios of *L1CAM* disruption ranged from 40% to 50% throughout this time-course study, *i*.*e*., from 4 to 14 days after transfection (Fig 3).

For comparison, we also conducted a similar FCM-based assay after disrupting *PIGA*. In this assay, SK-N-BE(2) cells were transfected with a Cas9 nuclease targeted to *PIGA* exon 6, stained with FLAER (an Alexa Fluor 488-labeled reagent that binds to GPI-anchor) [24, 25], and then FCM-analyzed on the same schedule as the *L1CAM* assay described above. This *PIGA*-based assay demonstrated that the ratio of FLAER-negative cells was low for 4 days after transfection but gradually increased until 14 days after transfection (Fig 3). These data collectively indicate that a minimum of 14 days of incubation is needed before conducting FCM analysis to accurately quantify *PIGA*-disrupted SK-N-BE(2) cells, whereas an FCM-based quantification of *L1CAM*-disrupted SK-N-BE(2) cells can be conducted, at the earliest, four days after transfection with Cas9 nucleases.

### Correction of *L1CAM*-inactivating mutations via targeted knock-in can be monitored using FCM

**Fig 4:**
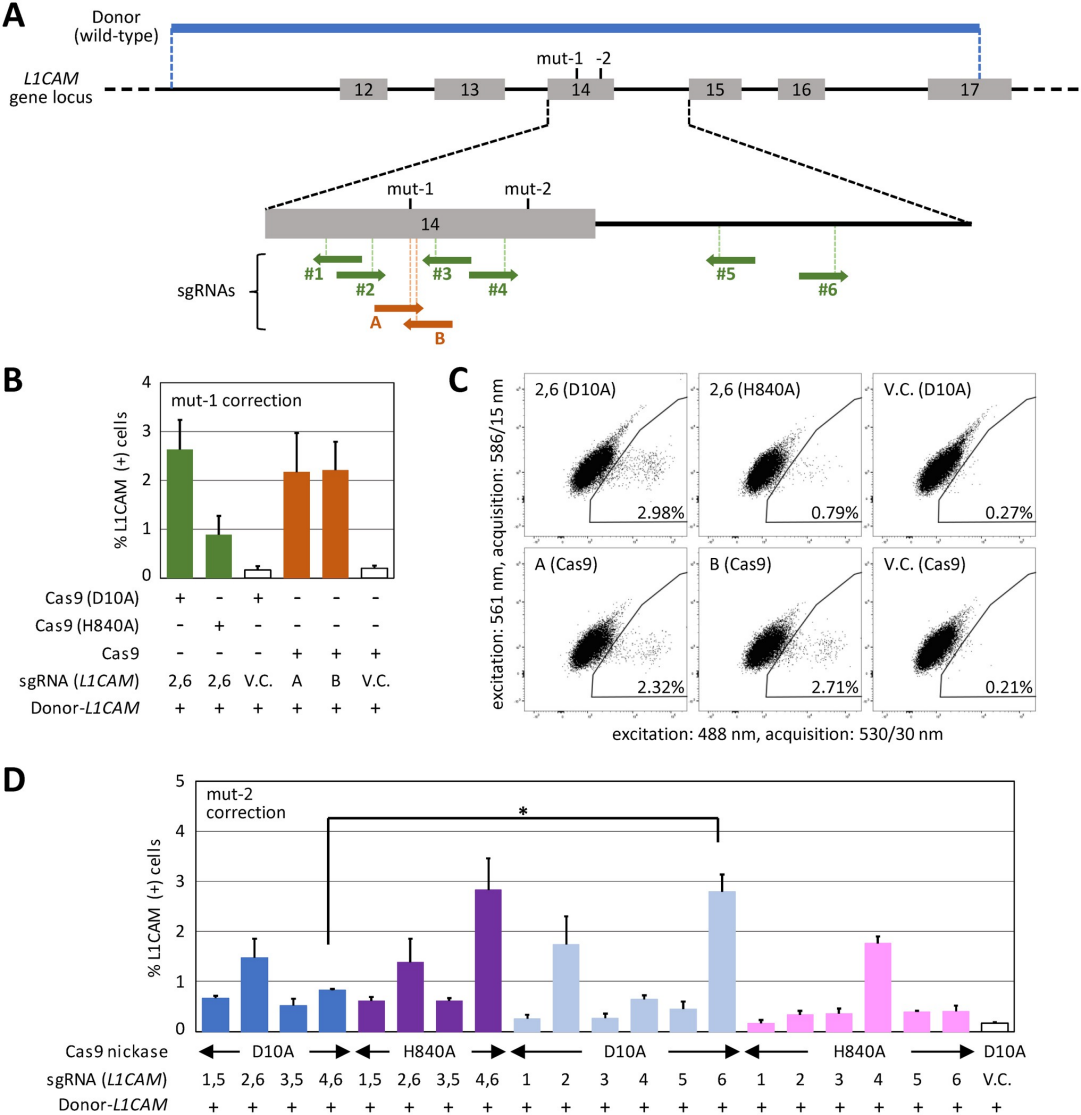
The efficiency of *L1CAM* gene correction can be quantified using FCM. **(A)** Schematic map of a genomic region surrounding *L1CAM* exon 14. Reporter clones harboring a truncating mutation within *L1CAM* exon 14 (mut-1 or mut-2) were established and used for gene correction assays. A 1,922-bp-long wild-type donor DNA covering a region from *L1CAM* intron 11 through exon 17 is displayed at the top. Shown at the bottom is an enlarged view of a region spanning exon 14 and intron 14, associated with the target sites of Cas9 nucleases (A and B) and Cas9 nickases (#1–#6). The arrows indicate the 5′ to 3′ direction of the Cas9 target sites. **(B)** Efficiency of mut-1 correction via Cas9 nuclease-based (brown) and tandem paired nicking (TPN)-based (green) targeted knock-in. The mut-1 reporter clone was transfected with a Cas9 nuclease loaded with sgRNA A or B, or D10A or H840A versions of Cas9 nickases loaded with sgRNAs #2 and #6, along with the wild-type donor. The cells were then FCM-analyzed to identify those that had become L1CAM-positive. Data represent the mean and SEM values from three independent experiments. **(C)** Representative dot plots obtained from the FCM analyses described in (B). The percentages of Alexa Fluor 488-positive cells are denoted in the plots. **(D)** The efficiency of mut-2 correction via TPN-based targeted knock-in. TPN was performed using the indicated pairs of sgRNAs together with Cas9 (D10A) (blue) or Cas9 (H840A) (purple), while a modified TPN procedure was conducted using single sgRNAs together with Cas9 (D10A) (light blue) or Cas9 (H840A) (pink). The cells were then FCM-analyzed to detect L1CAM-positive cells. Data represent the mean and SEM values from three independent experiments. * *p* < 0.001, significantly fewer L1CAM-positive cells were obtained using a sgRNA for TPN versus its use as a single sgRNA. Statistical analyses were performed using one-way ANOVA with post-hoc Bonferroni test.

We next explored the utility of the *L1CAM* assay to measure the efficiency of gene correction achieved via targeted knock-in. Initially, we created a reporter clone harboring a nonsense mutation in *L1CAM* exon 14 (mut-1 in Fig 4A, S6 and S7 Figs) from SK-N-BE(2) cells. This mutation was designed so that two protospacer adjacent motifs (PAMs; NGG) were located near or on the mutation and thus Cas9 nucleases using these PAMs (A and B) would cleave the mutant *L1CAM* in the reporter clone but not a wild-type donor plasmid. The developed reporter clone was then transfected with a Cas9 nuclease (A or B) along with the wild-type donor plasmid, allowing correction of the *L1CAM* mutation via targeted knock-in. Meanwhile, mut-1 in the reporter clone was also corrected via the TPN strategy [7] using a pair of Cas9 nickases targeted upstream and downstream of the mutated site (single guide RNAs (sgRNAs) #2 and #6). Cells undergoing targeted gene correction via Cas9 nuclease-based and TPN-based strategies were then subjected to fluorescent L1CAM labeling followed by FCM analysis, and the ratios of L1CAM-positive cells were determined. This FCM analysis showed that both Cas9 nuclease-based and TPN-based strategies for targeted knock-in effectively correct the *L1CAM* gene (Fig 4B and 4C). A similar level of *L1CAM* gene correction was achieved by the TPN strategy utilizing Cas9 (D10A) nickases and the Cas9 nuclease-based strategy, in line with our previous studies [7, 17, 26], although a rigorous comparison of knock-in efficiencies between these strategies is complicated due to the unavoidable use of different spacers in Cas9 nucleases and Cas9 nickases.

*L1CAM* is a pathogenic gene that, when mutated, causes various disorders including MASA syndrome (mental retardation, aphasia, shuffling gait, and adducted thumbs), X-linked hydrocephalus, spastic paraplegia, and corpus callosum agenesis [27]. We next chose a nonsense point mutation at exon 14 that had been found in multiple affected families [28] and introduced it into the endogenous *L1CAM* gene in SK-N-BE(2) cells (mut-2 in Fig 4A, S6 and S7 Figs). A cell clone was established from the resultant gene-engineered cells and utilized as a reporter to monitor the efficiency of targeted gene correction via TPN. In this analysis, Cas9 nickases (D10A and H840A versions) were used as tandem paired nickases or single nickases together with the wild-type donor plasmid. As a result, the mut-2 reporter clone proved useful in monitoring *L1CAM* gene correction via TPN, similar to the mut-1 reporter clone described above (Fig 4D). To verify this assay, Alexa Fluor 488-positive and -negative cell populations were sorted by FCM and genotyped for the mut-2 locus. It was then confirmed that the mut-2 mutation was reverted to the wild-type in Alexa Fluor 488-positive cells but was retained in Alexa Fluor 488-negative cells (S8 Fig). Consistent with our previous study [7], the efficiencies of gene correction achieved using single Cas9 nickases did not significantly exceed those achieved using tandem paired Cas9 nickases in both D10A and H840A versions (Fig 4D). However, there was an exception of sgRNA #6 coupled with Cas9 (D10A), suggesting that the effect of the TPN strategy may vary depending on experimental settings, or sgRNA #4 might introduce sporadic indels, disrupt *L1CAM*, and thereby compromise TPN-based gene correction by Cas9 (D10A) coupled with sgRNA #4 and #6. Overall, these data demonstrated that the *L1CAM* assay using *L1CAM*-mutant reporter cell clones as an assay platform can readily monitor the efficiency of targeted gene correction through simple procedures based on FCM. This gene correction assay is hereafter referred to as “*L1CAM* correction assay”.

### Correction of *L1CAM*-inactivating mutations via prime editing can be monitored using FCM

**Fig 5:**
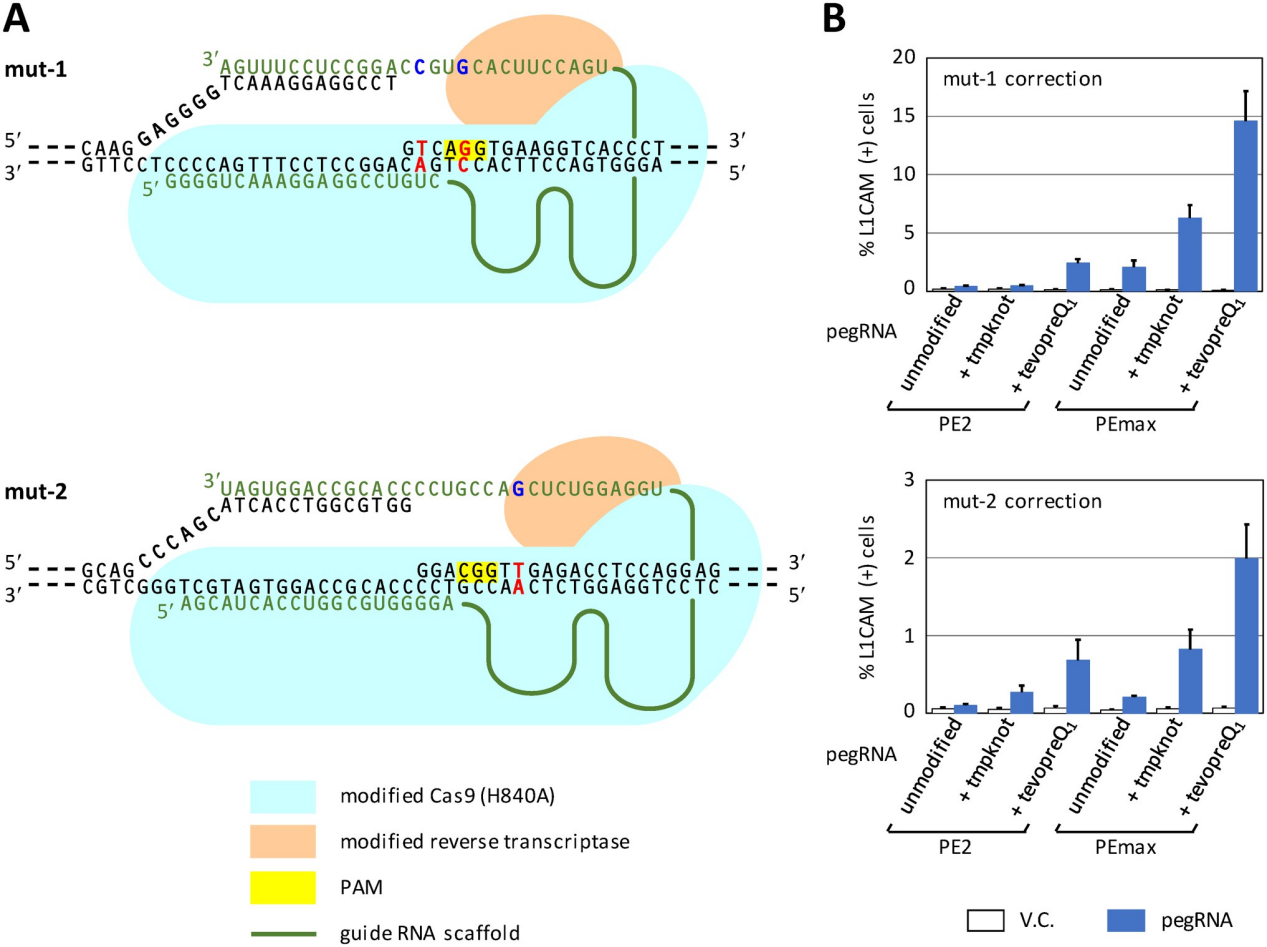
Correction of *L1CAM* mutations via prime editing can be quantified using FCM. **(A)** Schematic diagrams describing the correction of mut-1 (top) and mut-2 (bottom) *L1CAM* mutations via prime editing. Nucleotide sequences in black and green indicate gDNA and pegRNAs, respectively. Nucleotides shown by red and blue bold letters represent mutated genomic positions in SK-N-BE(2)-derived reporter clones and corresponding wild-type sequences in pegRNAs, respectively. Although not delineated in the diagrams, pegRNAs were either modified at their 3′ ends by appending “tmpknot” or “tevopreQ_1_” pseudoknot motifs [29], or left unmodified; *i*.*e*., three different types of pegRNAs for mut-1 and mut-2, respectively, were used in the assay. PAM, protospacer adjacent motif. See S1 Fig for the sequences of respective pegRNAs. **(B)** The efficiencies of mut-1 (top) and mut-2 (bottom) correction via prime editing. Prime editing was conducted using one of three pegRNAs (tmpknot, tevopreQ_1_, and unmodified) in combination with one of two prime editor proteins (PE2 and PEmax), and gene correction efficiencies achieved under the respective experimental settings were compared. PEmax is an enhanced version of the PE2 protein in which (i) additional amino acid changes were introduced in the Cas9 (H840A) domain to improve its nicking activity, (ii) the reverse transcriptase domain was codon-optimized for better expression in human cells, and (iii) additional nuclear localization signals were incorporated [30]. Data represent the mean and SEM values from three independent experiments. tmpknot, a trimmed pseudoknot from Moloney murine leukemia virus; tevopreQ_1_, a modified and trimmed prequeosine1-1 riboswitch aptamer [29].

We subsequently investigated whether the *L1CAM* correction assay could detect *L1CAM* gene correction achieved via prime editing [11]. We initially created a total of six plasmids expressing pegRNAs to correct *L1CAM* mutations (mut-1 and mut-2) in SK-N-BE(2)-derived reporter clones (Fig 5A). These plasmids were constructed with or without 3′-terminal modifications (tmpknot and tevopreQ_1_; S1 Fig) that increase pegRNA stability [29], and co-transfected into the reporter clones with one of the plasmids expressing differently modified Cas9 (H840A)-RT fusion proteins (PE2 and PEmax) [11, 30]. The subsequent FCM analyses showed a 2.0–5.1-fold higher efficiency of *L1CAM* gene correction using PEmax compared with the use of PE2, and 3′-terminal modifications (tmpknot and tevopreQ_1_) conferred 1.2–2.6-fold and 6.0–6.6-fold increases in *L1CAM* gene correction, respectively (Fig 5B). PEmax coupled with tevopreQ_1_ offered the highest gene correction efficiencies for both *L1CAM* mutations (19–36-fold increase compared with PE2 with unmodified pegRNA), among the plasmid combinations tested in this assay. The correction of mut-1 by prime editing was nearly one log more frequent than that of mut-2, potentially because mut-2, but not mut-1, is a single-base substitution, and DNA mismatch repair may impede the correction of mut-2 more severely than that of mut-1 [30]. Another potential reason for the different correction efficiencies between mut-1 and mut-2 may be that the correction of mut-1, but not mut-2, disrupts the PAM sequence and thus only the correction of mut-2 is interfered with by repeated nicking of edited alleles which might cause increased indel formation and decreased legitimate gene correction. Overall, these results are consistent with the findings obtained in previous studies [29, 30], supporting that the *L1CAM* correction assay quantitatively detects targeted knock-in elicited by prime editing.

### The *L1CAM* assay can be implemented using other antibodies, fluorophores, and cell lines

**Fig 6:**
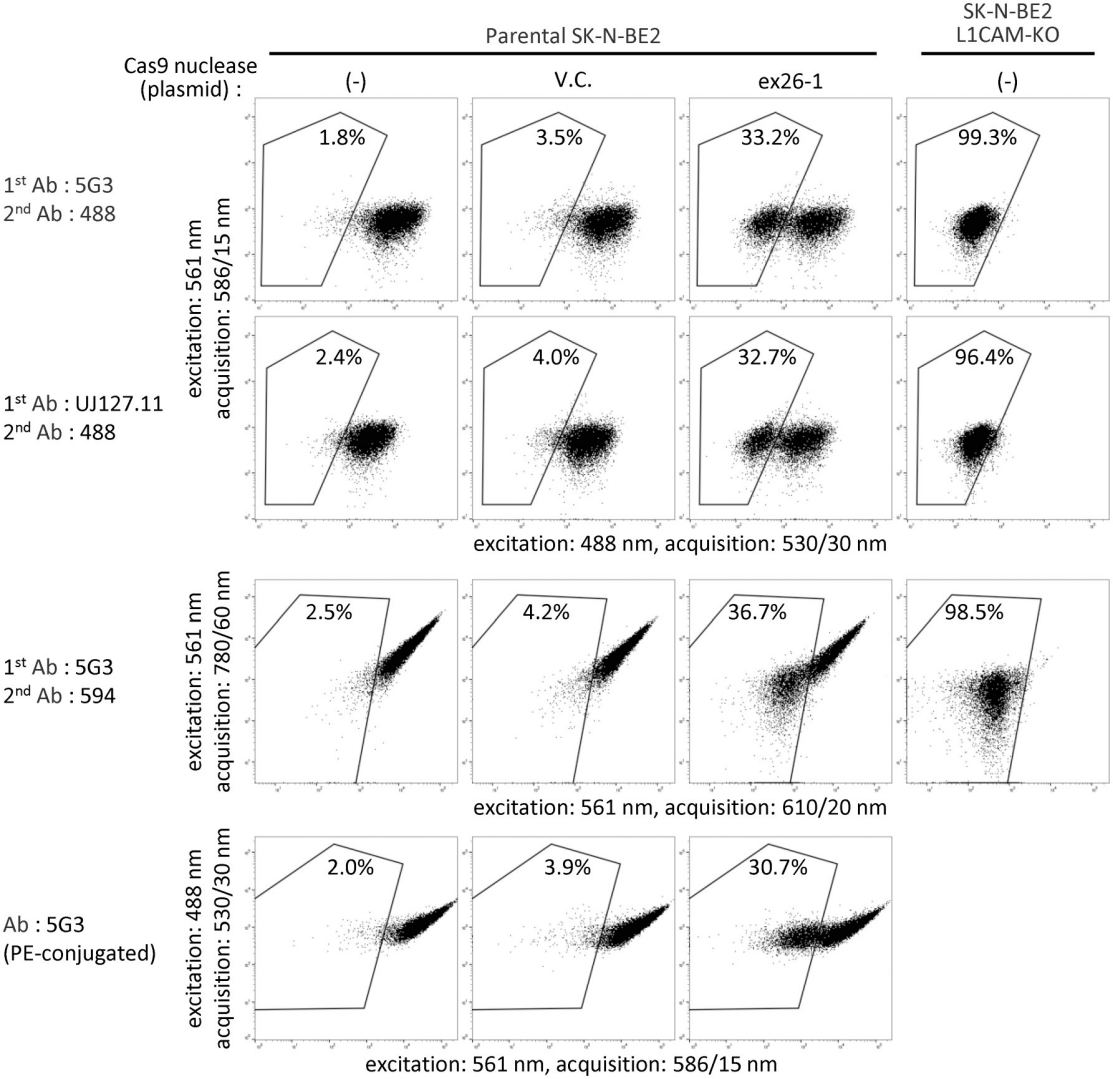
The *L1CAM* assay can be conducted using different antibodies, fluorophores, and FCM settings. SK-N-BE(2) cells were transfected with ex26-1 nuclease, a vector control, or none, and stained with antibodies indicated on the left. Cells were then analyzed by FCM using laser and filter settings shown along the X- and Y-axes of the dot plots. For an additional control, *L1CAM* knockout cells were included in this assay as displayed on the right. The *L1CAM* knockout cells were obtained via FCM-based sorting of *L1CAM*-negative cells from SK-N-BE(2) transfected with ex26-1 nuclease. Percentages of fluorescence-negative cells are denoted in the dot plots. UJ127.11, anti-L1CAM monoclonal antibody UJ127.11; 594, Alexa Fluor 594-conjugated secondary antibody; PE, phycoerythrin.

Although most *L1CAM* assays described above used 5G3 anti-L1CAM antibody and Alexa Fluor 488-conjugated secondary antibody, it should be possible to differentiate between L1CAM-positive and -negative cells using other antibodies and fluorophores for FCM. To prove this, we used UJ127.11, another antibody detecting the extracellular portion of L1CAM [31], instead of 5G3, and Alexa Fluor 594 and PE as fluorophores bound to antibodies. As shown in Fig 6, we successfully detected *L1CAM*-disrupted SK-N-BE(2) cells in assays employing these reagents by selecting appropriate laser wavelengths and filters in FCM.

**Fig 7:**
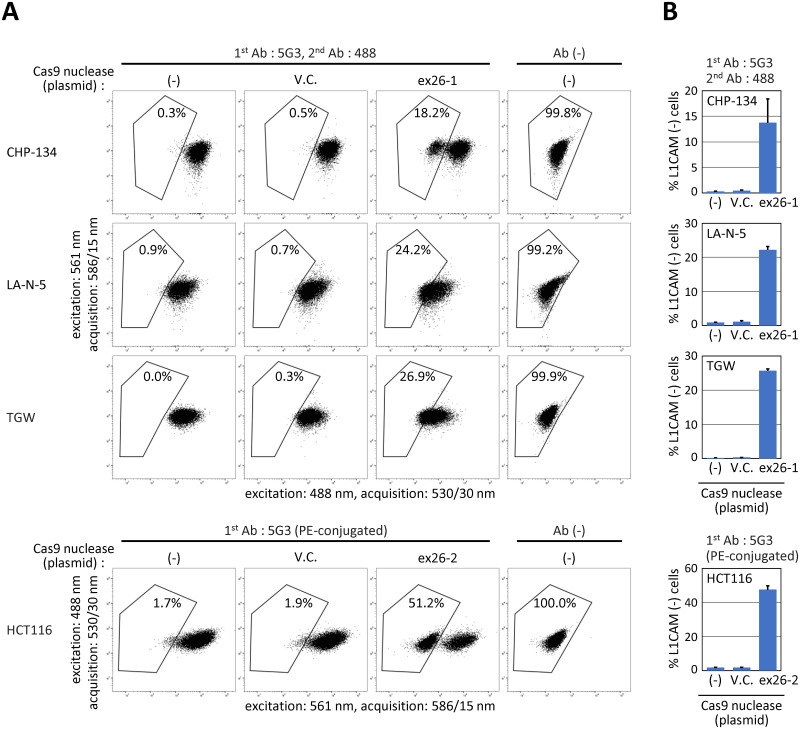
Near-diploid male cell lines highly expressing *L1CAM*, besides SK-N-BE(2), may serve as platforms for the *L1CAM* assay. **(A)** Representative dot plots obtained in this analysis. The CHP-134, LA-N-5, and TGW neuroblastoma cell lines and the HCT116 colon cancer cell line were transfected with ex26-1 or ex26-2 nuclease, a vector control, or none. The cells were then stained as indicated at the top of the panels and analyzed applying FCM settings noted along the X- and Y-axes of dot plots. Percentages of Alexa Fluor 488-negative cells are denoted in the plots. **(B)** Graphical summary of the experimental results, with representatives shown in (A). The results of FCM analyses conducted without staining cells are omitted from the graphs. Data represent the mean and SEM values from three independent experiments.

In addition, we disrupted *L1CAM* in three more neuroblastoma cell lines derived from male patients (CHP-134, LA-N-5, and TGW) that robustly express *L1CAM*. Fluorescent L1CAM labeling followed by FCM showed that *L1CAM*-disrupted cells were well separated from *L1CAM*-intact cells on dot plots in CHP-134 (Fig 7). In assays with LA-N-5 and TGW, *L1CAM*-disrupted and *L1CAM*-intact cells were not clearly distinguished, although a subset of cells likely became L1CAM-negative as indicated by the leftward shift of cell populations on the dot plots.

Robust *L1CAM* expression is not exclusive to neuroblastoma cell lines. We asked whether the *L1CAM* assay could be conducted with HCT116, a colon cancer cell line with robust *L1CAM* expression. It was found that *L1CAM*-disrupted HCT116 cells were clearly separated from their *L1CAM*-positive counterparts on a dot plot, similar to SK-N-BE(2) and CHP-134 (Fig 7). These data indicate that various human cell lines can be used as platforms for the *L1CAM* assay.

## Discussion

Genome editing events can be monitored in “live” diploid male cells using simple FCM-based procedures, provided that the edited gene (i) is located on the X-chromosome (outside pseudoautosomal regions), (ii) encodes a cell surface protein or a protein needed for producing or transporting cell surface molecules such as GPI-anchors, (iii) has a reliable antibody or reagent that specifically binds to the extracellular portion of the protein, (iv) is constitutively expressed in a platform cell line, and (v) has no significant impact on the growth of the platform cell line. Genome-editing assays performed using such target genes help researchers readily compare genome editing efficiencies achieved using different methods and improve genome editing technologies. The “live” cells analyzed in the assay can subsequently undergo FCM-based sorting, clonal isolation of gene-edited cells, assessments for genetic changes created, and investigation on the biological consequences of the edits if desired. In this study, we postulated that the *L1CAM* gene located on Xq28 (outside the pseudoautosomal region) may be among rare genes that fulfill the above-mentioned criteria, and addressed its utility as a target gene in genome-editing assays. As a result, we demonstrated that simple yet high-throughput FCM-based cell analysis in the *L1CAM* assay allows for sensitive and quantitative detection of knockouts and knock-ins induced by Cas9 nucleases and nickases.

*L1CAM* encodes a single-pass membrane protein distributed over the cell surface [19, 20]. Our experimental results suggest that the target sequences of Cas9 nucleases used in the *L1CAM* assay can be widely selected from exons encoding the L1CAM extracellular domain. In addition, the *L1CAM* assay can be performed under various experimental conditions, including the use of alternative antibodies, fluorophores, laser wavelengths, and FCM filters. In this study, we used two commercially available monoclonal antibodies that recognize the L1CAM extracellular domain and demonstrated their utility in the *L1CAM* assay.

*L1CAM* is highly expressed in the central nervous system, and the large intestine is one of the organs exhibiting moderate *L1CAM* expression, according to the Expression Atlas database. Consistent with this expression pattern, we found that four neuroblastoma cell lines and a colon cancer cell line, HCT116, exhibit high *L1CAM* expression levels. We thus attempted to apply these cell lines to the *L1CAM* assay and demonstrated that SK-N-BE(2), CHP-134, and HCT116 are appropriate for platform cell lines in this assay. To the best of our knowledge, this study is the first to use neuroblastoma cell lines as platforms for FCM-based assays to quantify genome editing efficiencies.

Because the *L1CAM* assay is independent of the GPI-anchor biosynthesis pathway, this assay remains functional even when the GPI-anchor biosynthesis pathway is impaired in platform cells. This implies that, unlike *PIGA*, *PIGP*, and *CD55*-based assays, the *L1CAM* assay can be conducted using platform cell lines that have alterations or loss of expression in one of the many GPI-anchor biosynthesis genes. We also demonstrated that the *L1CAM* assay takes less time to evaluate knockout than the *PIGA* assay. This is likely because the loss of GPI-anchors upon *PIGA* disruption occurs through the abolition of a relatively complicated molecular chain reaction; *PIGA* disruption initially causes a gradual reduction in PIGA protein levels because of degradation, followed by the cessation of GPI-anchor biosynthesis and, finally, the loss of GPI-anchors from the cell surface [32]. In contrast, the *L1CAM* assay operates through a simple mechanism; the loss of L1CAM protein from the cell surface directly results from *L1CAM* disruption.

*L1CAM* is a causative gene for a spectrum of hereditary genetic disorders including MASA syndrome [27]. We created a reporter clone for the *L1CAM* correction assay by knocking-in a pathogenic *L1CAM* mutation discovered in affected families into SK-N-BE(2) cells. We then employed the reporter clone to assess the efficiency of *L1CAM* gene correction. The methodologies and cell clones established in these experiments may be useful in the future to investigate the pathobiology caused by *L1CAM* mutations.

In summary, we developed an FCM-based assay using *L1CAM* as a reporter gene and demonstrated its utility in measuring the efficiencies of knockout and targeted knock-in. This assay will help researchers further improve the current genome editing methodologies and develop new technologies for highly efficient targeted knock-in. In addition, more genome-editing assays may be established by adopting the same approach to other X-chromosomal genes that fulfill the criteria mentioned above, which will provide further options to achieve sensitive and convenient measurement of genome editing efficiencies.

## Supporting information

S1 Fig Structure and DNA sequences of synthetic transcription cassettes within plasmids expressing unmodified and modified pegRNAs. **(A)** Schematic diagrams of synthetic transcription cassettes expressing pegRNAs and epegRNAs (tmpknot and tevopreQ_1_) targeted to mut-1, mut-2, and non-target controls (V.C.). Genetic components depicted with color squares are connected without intervening sequences in the plasmids indicated on the left. **(B)** DNA sequences of the genetic components depicted in (A). Nucleotides indicated by red and blue bold letters represent mutant and wild-type sequences at the edited positions, respectively. Underlining indicates restriction enzyme recognition sites. The lowercase letter “g” indicates a guanine appended to the 5′ end of mut-2 spacer for enhanced transcription. epegRNA, engineered prime editing guide RNA; RTT, reverse transcription template; PBS, primer binding site.
(PDF)

S2 Fig A 4,825-bp-long DNA sequence of Donor-*L1CAM*. Yellow and blue shading indicates a 1,922-bp-long donor DNA fragment and *L1CAM* exon 14 within the donor sequence, respectively. Letters with purple and red backgrounds represent wild-type sequences corresponding to mut-1 and mut-2 sites, respectively. Nucleotide sequences without shading represent those from pBluescript II KS (+). See S6 and S7 Figs for the sequences of mut-1 and mut-2.
(PDF)

S3 Fig The mut-1 and mut-2 reporter clones and their parental SK-N-BE(2) cell line exhibit similar proliferation indices. Proliferation indices of the cells listed on the right were assessed by MTT assay. Cell numbers at 24–120 h after cell seeding were evaluated every 24 h and are indicated relative to those at 24 h after cell seeding. Data represent the mean and SEM values from three independent experiments. Quadruplicate samples were analyzed in each experiment.
(PDF)

S4 Fig Alexa Fluor 488 negativity in the *L1CAM* assay represents *L1CAM* disruption by indels. SK-N-BE(2) cells were transfected with a Cas9 nuclease targeted to *L1CAM* (ex26-1), and the L1CAM protein on the surface of transfected cells was labeled with Alexa Fluor 488. Cells were then FCM-sorted to isolate Alexa Fluor 488-positive and -negative cells. Next, a genomic region spanning the edited site at the *L1CAM* intron 25–exon 26 boundary was PCR-amplified using gDNAs extracted from the Alexa Fluor 488-positive and -negative cells as templates. Lastly, the amplified PCR products were cloned into a plasmid, and multiple plasmids containing the PCR products as inserts were isolated and sequenced. **(A)** DNA sequences of PCR products amplified from Alexa Fluor 488-positive cells. Sequences are shown in alignment with a wild-type control derived from parental SK-N-BE(2) cells displayed at the top. A red letter indicates an inserted nucleotide. **(B)** Representative sequencing chromatogram obtained in the analysis shown in (A). **(C)** DNA sequences of PCR products amplified from Alexa Fluor 488-negative cells displayed in a manner similar to (A). A green letter indicates a substituted nucleotide. **(D)** Two representative sequencing chromatograms obtained in the analysis shown in (C). **(E)** *L1CAM* genotypes in Alexa Fluor 488-positive and -negative cells determined based on the experimental results shown in (A)–(D). In (A)–(D), the vertical dotted lines in red indicate a genomic site cleaved by ex26-1. WT, wild-type; ins, insertion; del, deletion; subst, substitution.
(PDF)

S5 Fig *L1CAM* exon 3 is an alternative exon. **(A)** *L1CAM* exons 2–4 were amplified using cDNA from SK-N-BE(2) as a template, and the resultant bulk PCR product was Sanger sequenced. Top, a scheme of *L1CAM* exons 1–5 associated with primers used for PCR (green arrows). Bottom, sequencing chromatograms obtained using forward (F) and reverse (R) sequencing primes. Annotations “mixed” indicate that an intermixture of multiple sequences initiates immediately after the terminus of *L1CAM* exon 2 (F) and exon 4 (R), consistent with the alternative splicing of exon 3. **(B)** The bulk PCR product obtained in (A) was fractionated on an agarose gel as shown on the left. Two distinct PCR products of approximately 300-bp in size were isolated from the gel and Sanger sequenced using a forward sequencing primer. Shown on the right are two chromatograms, one exhibiting a sequence of *L1CAM* exons 2–4 and the other revealing joined sequences of *L1CAM* exons 2 and 4. Primers used for PCR amplification and Sanger sequencing are listed in S3 Table.
(PDF)

S6 Fig DNA sequences surrounding the mutated genomic sites at *L1CAM* exon 14 in the SK-N-BE(2)-derived mut-1 and mut-2 reporter clones (top) and the corresponding wild-type sequence in the Donor-*L1CAM* plasmid (bottom). Target sequences of Cas9 nucleases (A and B, brown) and Cas9 nickases (#1–#6, green) are color-shaded, with darker shading on the neighboring PAMs. Brown and green numbers are arbitrary values showing the relative positions of cleavage by Cas9 nucleases and nickases, respectively. Nucleotides indicated by bold letters with pink and blue shading represent mut-1 and mut-2 truncating mutations in the reporter clones and their corresponding wild-type sequences in Donor-*L1CAM*, respectively. Uppercase and lowercase letters in DNA sequences indicate exonic and intronic sequences, respectively.
(PDF)

S7 Fig Sequence chromatograms showing mutated genomic sites in the mut-1 and mut-2 reporter clones and the corresponding genomic sites in the parental SK-N-BE(2) cell line. Letters with pink and blue shading indicate mutated and wild-type sequences, respectively.
(PDF)

S8 Fig Alexa Fluor 488 positivity in the *L1CAM* correction assay represents the reversion of *L1CAM* mut-2 to a wild-type sequence. The SK-N-BE(2)-derived mut-2 reporter clone was transfected with sgRNA #4 and #6 coupled with Cas9 (H840A) and Donor-*L1CAM* to correct the *L1CAM* mutation via TPN. The L1CAM protein on the surface of transfected cells was labeled with Alexa Fluor 488, and cells were subjected to FCM-based sorting to isolate Alexa Fluor 488-positive and -negative populations. PCR was then performed to amplify a genomic region spanning the mut-2 site within *L1CAM* exon 14 in the Alexa Fluor 488-positive and -negative populations. The amplified PCR products were cloned into a plasmid, and multiple plasmids containing the PCR products as inserts were isolated and sequenced. **(A)** DNA sequences of PCR products amplified from Alexa Fluor 488-positive cells. Sequences are shown in alignment with a wild-type control derived from parental SK-N-BE(2) cells displayed at the top. Arbitrary numbers placed above the aligned sequences indicate the relative positions of nucleotides. Blue shading indicates a wild-type sequence resulting from mut-2 reversion. Green letters indicate substituted nucleotides. **(B)** Representative sequencing chromatogram obtained in the analysis shown in (A). **(C)** DNA sequences of PCR products amplified from Alexa Fluor 488-negative cells displayed in a manner similar to (A). Red shading indicates the mut-2 nonsense mutation. **(D)** Representative sequencing chromatogram obtained in the analysis shown in (C). **(E)** *L1CAM* genotypes in Alexa Fluor 488-positive and -negative cells determined based on the experimental results shown in (A)–(D). 1-bp substitutions shown in (A) and (C), probably introduced during genome editing or by PCR errors, are not considered in genotyping *L1CAM*, because they are located within an intronic sequence distant from the exon–intron boundary. In (A)–(D), the vertical dotted lines in red indicate a genomic site nicked by Cas9 (H840A) coupled with sgRNA #4 or #6. WT, wild-type; mut, mutant.
(PDF)

S1 Table Cas9 target sites with PAMs. (PDF)

S2 Table Potential off-target sites for the sgRNAs used in this study. (XLSX)

S3 Table Primer sequences. (PDF)
