# Supplementary material for: Flow cytometry-based quantification of genome editing efficiency in human cell lines using the L1CAM gene
Source: PLoS One. 2023 Nov 9;18(11):e0294146. doi: 10.1371/journal.pone.0294146 (PMC10635454; doi:10.1371/journal.pone.0294146)
Supplement: S1 Fig — (A) Schematic diagrams of synthetic transcription cassettes expressing pegRNAs and epegRNAs (tmpknot and tevopreQ1) targeted to mut-1, mut-2, and non-target controls (V.C.). Genetic components depicted with color squares are connected without intervening sequences in the plasmids indicated on the left. (B) DNA sequences of the genetic components depicted in (A). Nucleotides indicated by red and blue bold letters represent mutant and wild-type sequences at the edited positions, respectively. Underlining indicates restriction enzyme recognition sites. The lowercase letter “g” indicates a guanine appended to the 5′ end of mut-2 spacer for enhanced transcription. epegRNA, engineered prime editing guide RNA; RTT, reverse transcription template; PBS, primer binding site. (PDF) [file pone.0294146.s001.pdf]

S1 Fig

A

	5′	spacer		RTT-PBS	3′-tail 3′
L1CAM.pegRNA-1	U6 promoter	mut-1	gRNA scaffold	mut-1	polyT
L1CAM.epegRNA(tmpknot)-1	U6 promoter	mut-1	gRNA scaffold	mut-1	tmpknot-polyT
L1CAM.epegRNA(tevopreQ$_1$)-1	U6 promoter	mut-1	gRNA scaffold	mut-1	tevopreQ$_1$-polyT
L1CAM.pegRNA-2	U6 promoter	mut-2	gRNA scaffold	mut-2	polyT
L1CAM.epegRNA(tmpknot)-2	U6 promoter	mut-2	gRNA scaffold	mut-2	tmpknot-polyT
L1CAM.epegRNA(tevopreQ$_1$)-2	U6 promoter	mut-2	gRNA scaffold	mut-2	tevopreQ$_1$-polyT
pegRNA-V.C.	U6 promoter	BbsI-BbsI	gRNA scaffold	BsmBI-BsmBI	polyT
epegRNA (tmpknot)-V.C.	U6 promoter	BbsI-BbsI	gRNA scaffold	BsmBI-BsmBI	tmpknot-polyT
epegRNA (tevopreQ$_1$)-V.C.	U6 promoter	BbsI-BbsI	gRNA scaffold	BsmBI-BsmBI	tevopreQ$_1$-polyT

B

U6 promoter		5′- - - - GTGGAAAGGACGAAACACC
spacer	mut-1	GGGGTCAAAGGAGGCCTGTC
	mut-2	gAGCATCACCTGGCGTGGGGA
	BbsI-BbsI	GGGTCTTCGAGAAGACCT
gRNA scaffold		GTTTTAGAGCTAGAAATAGCAAGTTAAAATAAGGCTAG TCCGTTATCAACTTGAAAAAGTGGCACCGAGTCGGTGC
RTT-PBS	mut-1	TGACCTTCACGTGCCAGGCCTCCTTTGA
	mut-2	TGGAGGTCTCGACCGTCCCCACGCCAGGTGAT
	BsmBI-BsmBI	TGAGACGATCAATTCCGTCTCA
3′-tail	polyT	TTTTTTT - - - -3′
	tmpknot-polyT	TCAGGAGCCCCCCCCCTGAACCCAGGATAACCCTCAAA GTCGGGGGGCTTTTTTT - - - -3′
	tevopreQ$_1$-polyT	CGCGGTTCTATCTAGTTACGCGTTAAACCAACTAGAATT TTTTT - - - -3′

S1 Fig. Structure and DNA sequences of synthetic transcription cassettes within plasmids expressing unmodified and modified pegRNAs.
(A) Schematic diagrams of synthetic transcription cassettes expressing pegRNAs and epegRNAs (tmpknot and tevopreQ$_1$) targeted to mut-1, mut-2, and non-target controls (V.C.). Genetic components depicted with color squares are connected without intervening sequences in the plasmids indicated on the left.
(B) DNA sequences of the genetic components depicted in (A). Nucleotides indicated by red and blue bold letters represent mutant and wild-type sequences at the edited positions, respectively. Underlining indicates restriction enzyme recognition sites. The lowercase letter “g” indicates a guanine appended to the 5' end of mut-2 spacer for enhanced transcription. epegRNA, engineered prime editing guide RNA; RTT, reverse transcription template; PBS, primer binding site.
