# Supplementary material for: Flow cytometry-based quantification of genome editing efficiency in human cell lines using the L1CAM gene
Source: PLoS One. 2023 Nov 9;18(11):e0294146. doi: 10.1371/journal.pone.0294146 (PMC10635454; doi:10.1371/journal.pone.0294146)
Supplement: S2 Fig — Yellow and blue shading indicates a 1,922-bp-long donor DNA fragment and L1CAM exon 14 within the donor sequence, respectively. Letters with purple and red backgrounds represent wild-type sequences corresponding to mut-1 and mut-2 sites, respectively. Nucleotide sequences without shading represent those from pBluescript II KS (+). See S6 and S7 Figs for the sequences of mut-1 and mut-2. (PDF) [file pone.0294146.s002.pdf]

S2 Fig

GTGGCACTTTTCGGGGAAATGTGCGCGGAACCCCTATTTGTTTATTTTTCTAAATACATTCAAATATGTATCCGCTCATGAGACAATAACCCTGATAAAT
GCTTCAATAATATTGAAAAAGGAAGAGTATGAGTATTCAACATTTCCGTGTCGCCCTTATTCCCTTTTTTGCGGCATTTTGCCTTCCTGTTTTTGCTCAC
CCAGAAACGCTGGTGAAAGTAAAAGATGCTGAAGATCAGTTGGGTGCACGAGTGGGTTACATCGAACTGGATCTCAACAGCGGTAAGATCCTTGAGAGTT
TTCGCCCCGAAGAACGTTTTCCAATGATGAGCACTTTTAAAGTTCTGCTATGTGGCGCGGTATTATCCCGTATTGACGCCGGGCAAGAGCAACTCGGTCG
CCGCATACACTATTCTCAGAATGACTTGGTTGAGTACTCACCAGTCACAGAAAAGCATCTTACGGATGGCATGACAGTAAGAGAATTATGCAGTGCTGCC
ATAACCATGAGTGATAACACTGCGGCCAACTTACTTCTGACAACGATCGGAGGACCGAAGGAGCTAACCGCTTTTTTGCACAACATGGGGGATCATGTAA
CTCGCCTTGATCGTTGGGAACCGGAGCTGAATGAAGCCATACCAAACGACGAGCGTGACACCACGATGCCTGTAGCAATGGCAACAACGTTGCGCAAACT
ATTAACTGGCGAACTACTTACTCTAGCTTCCCGGCAACAATTAATAGACTGGATGGAGGCGGATAAAGTTGCAGGACCACTTCTGCGCTCGGCCCTTCCG
GCTGGCTGGTTTATTGCTGATAAATCTGGAGCCGGTGAGCGTGGGTCTCGCGGTATCATTGCAGCACTGGGGCCAGATGGTAAGCCCTCCCGTATCGTAG
TTATCTACACGACGGGGAGTCAGGCAACTATGGATGAACGAAATAGACAGATCGCTGAGATAGGTGCCTCACTGATTAAGCATTGGTAACTGTCAGACCA
AGTTTACTCATATATACTTTAGATTGATTTAAAACTTCATTTTTAATTTAAAAGGATCTAGGTGAAGATCCTTTTTGATAATCTCATGACCAAAATCCCT
TAACGTGAGTTTTCGTTCCACTGAGCGTCAGACCCCGTAGAAAAGATCAAAGGATCTTCTTGAGATCCTTTTTTTCTGCGCGTAATCTGCTGCTTGCAAA
CAAAAAAACCACCGCTACCAGCGGTGGTTTGTTTGCCGGATCAAGAGCTACCAACTCTTTTTCCGAAGGTAACTGGCTTCAGCAGAGCGCAGATACCAAA
TACTGTCCTTCTAGTGTAGCCGTAGTTAGGCCACCACTTCAAGAACTCTGTAGCACCGCCTACATACCTCGCTCTGCTAATCCTGTTACCAGTGGCTGCT
GCCAGTGGCGATAAGTCGTGTCTTACCGGGTTGGACTCAAGACGATAGTTACCGGATAAGGCGCAGCGGTCGGGCTGAACGGGGGGTTCGTGCACACAGC
CCAGCTTGGAGCGAACGACCTACACCGAACTGAGATACCTACAGCGTGAGCTATGAGAAAGCGCCACGCTTCCCGAAGGGAGAAAGGCGGACAGGTATCC
GGTAAGCGGCAGGGTCGGAACAGGAGAGCGCACGAGGGAGCTTCCAGGGGGAAACGCCTGGTATCTTTATAGTCCTGTCGGGTTTCGCCACCTCTGACTT
GAGCGTCGATTTTTGTGATGCTCGTCAGGGGGGCGGAGCCTATGGAAAAACGCCAGCAACGCGGCCTTTTTACGGTTCCTGGCCTTTTGCTGGCCTTTTG
CTCACATGTTCTTTCCTGCGTTATCCCCTGATTCTGTGGATAACCGTATTACCGCCTTTGAGTGAGCTGATACCGCTCGCCGCAGCCGAACGACCGAGCG
CAGCGAGTCAGTGAGCGAGGAAGCGGAAGAGCGCCCAATACGCAAACCGCCTCTCCCCGCGCGTTGGCCGATTCATTAATGCAGCTGGCACGACAGGTTT
CCCGACTGGAAAGCGGGCAGTGAGCGCAACGCAATTAATGTGAGTTAGCTCACTCATTAGGCACCCCAGGCTTTACACTTTATGCTTCCGGCTCGTATGT
TGTGTGGAATTGTGAGCGGATAACAATTTCACACAGGAAACAGCTATGACCATGATTACGCCAAGCGCGCAATTAACCCTCACTAAAGGGAACAAAAGCT
GGGTACCGGGCCCCCCCTCGAGCGATAGCCGAGGGAGATGTAAGAGGGGAAAGGTCTTGTCACTGTGACTGAGCGGACCGCTGTGCCACAGGATGTGACC
AAGGCCAGCCCAGGTGGCACCTCTCCTGGAACTTCAGGGAAGGCTGGGGACTAGGCCAGGAAGGCTTCTGGGGAGAGGTGACTGTCAGTTAGGCCTGGAA
TAACGTAAAAGTTGGGTGGCTGTGACCAGAGGCGCATCTGAGGCAGAGGGCAGTGTCTGAAGGCAGAGAGAGACACAGCCTGGCGGGGGCCTCAGCTGGG
AGCAGGGAAGCAAGATTTGCAGGGCTCTTGGGGGCCCGAGGAAGCCGTGCAGAGGGTCCCTGGCTCCTGGCCCAGCTGTGGTCCCAAGTCCTGCCCTGTC
TCCTGAGGCTGTTCCTCCCCTAGAGCTGCCAGCCAAGATCCTGACTGCGGACAATCAGACGTACATGGCTGTCCAGGGCAGCACTGCCTACCTTCTGTGC
AAGGCCTTCGGAGCGCCTGTGCCCAGTGTTCAGTGGTGAGTGTCTCGTCCTGGTAGTGGTGAGTGTCGTGTCCCAGTGGCCAGGGAGCCAGGGAGGGCAG
GGAGCCCAGGCCAGCCAGTCAGAGCCAGGCCCGCCCTGCTCCCTCCAGGCTGGACGAGGATGGGACAACAGTGCTTCAGGACGAACGCTTCTTCCCCTAT
GCCAATGGGACCCTGGGCATTCGAGACCTCCAGGCCAATGACACCGGACGCTACTTCTGCCTGGCTGCCAATGACCAAAACAATGTTACCATCATGGCTA
ACCTGAAGGTTAAAGGTCAGGCAACCCTTGCCACATGGCTGGCAGTGCCTCTGGGAGGGAGGGTCTGGGCCTCTGGAGGACAACAGAGTGACTTCCCCAC
GCACGCATTACCCTCAGATGCAACTCAGATCACTCAGGGGCCCCGCAGCACAATCGAGAAGAAAGGTTCCAGGGTGACCTTCACGTGCCAGGCCTCCTTT
GACCCCTCCTTGCAGCCCAGCATCACCTGGCGTGGGGACGGTCGAGACCTCCAGGAGCTTGGGGACAGTGACAAGTGAGGACAGTGACGGTGAAAGGGGG
CAGAGTGGGAAAAGCTGGAAGTCCAGACCTCTTGGCCTCGTCCTTGCTTTGATGGGGAAGGCTCTGACAGGAGCGGGGGAAGGAGACAGGAGGGAGGGAT
GGGAGGGGGAGAAGAGCCCAGATGGCAGGAAAGACAGACACGGCTCCTCCGCAGGTACTTCATAGAGGATGGGCGCCTGGTCATCCACAGCCTGGACTACA
GCGACCAGGGCAACTACAGCTGCGTGGCCAGTACCGAACTGGATGTGGTGGAGAGTAGGGCACAGCTCTTGGTGGTGGGTAAGTCCTAGGGTGGAAGCCT
CCACTCCAGAAGGCCGGGGCTCACATCCCTGGGCCCTTTCAAGCACCGACCCTCCCCACCCTCAGGGAGCCCTGGGCCGGTGCCACGGCTGGTGCTGTCC
GACCTGCACCTGCTGACGCAGAGCCAGGTGCGCGTGTCCTGGAGTCCTGCAGAAGACCACAATGCCCCCATTGAGAGTAAGAGGCTTGAGCTCAGTGCCA
CCCCACCGTCACTTCTGCCAGCCCTGGCCCCTCCTGGGGTCCTCCCTCCTCCTGGAGCCTTCCTGGGGGGACACGGTTTGGGGGGACTCACGGTTCCTCA
TGGGAATCTGGAGATGCCAGTTGGCCTGGGTAATCAGGGACCCAGACTGTACCCAACCCCTCCACAGCCCTTCCCCCAAAGCCACATGCTGATCACTCCA
TTGTCGGTTCATTTCTTGGCAGAATATGACATTGAATTTGAGGACAAGGAAATGGCGCCTGAAAAATGGTACAGTCTGGGCAAGGTTCCAGGGAACCAGA
CCTCTACCACCCTCAAGCTGTCGCCCTATGTCCACTACACCTTTCTAGAGCGGCCGCCACCGCGGTGGAGCTCCAATTCGCCCTATAGTGAGTCGTATTA
CGCGCGCTCACTGGCCGTCGTTTTACAACGTCGTGACTGGGAAAACCCTGGCGTTACCCAACTTAATCGCCTTGCAGCACATCCCCCTTTCGCCAGCTGG
CGTAATAGCGAAGAGGCCCGCACCGATCGCCCTTCCCAACAGTTGCGCAGCCTGAATGGCGAATGGGACGCGCCCTGTAGCGGCGCATTAAGCGCGGCGG
GTGTGGTGGTTACGCGCAGCGTGACCGCTACACTTGCCAGCGCCCTAGCGCCCGCTCCTTTCGCTTTCTTCCCTTCCTTTCTCGCCACGTTCGCCGGCTT
TCCCCGTCAAGCTCTAAATCGGGGGCTCCCTTTAGGGTTCCGATTTAGTGCTTTACGGCACCTCGACCCCAAAAAACTTGATTAGGGTGATGGTTCACGT
AGTGGGCCATCGCCCTGATAGACGGTTTTTCGCCCTTTGACGTTGGAGTCCACGTTCTTTAATAGTGGACTCTTGTTCCAAACTGGAACAACACTCAACC
CTATCTCGGTCTATTCTTTTGATTTATAAGGGATTTTGCCGATTTCGGCCTATTGGTTAAAAAATGAGCTGATTTAACAAAAATTTAACGCGAATTTTAA
CAAAATATTAACGCTTACAATTTAG

S2 Fig. A 4,825-bp-long DNA sequence of Donor-*L1CAM*. Yellow and blue shading indicates a 1,922-bp-long donor DNA fragment and *L1CAM* exon 14 within the donor sequence, respectively. Letters with purple and red backgrounds represent wild-type sequences corresponding to mut-1 and mut-2 sites, respectively. Nucleotide sequences without shading represent those from pBluescript II KS (+). See S6 Fig and S7 Fig for the sequences of mut-1 and mut-2.
