# Supplementary material for: Flow cytometry-based quantification of genome editing efficiency in human cell lines using the L1CAM gene
Source: PLoS One. 2023 Nov 9;18(11):e0294146. doi: 10.1371/journal.pone.0294146 (PMC10635454; doi:10.1371/journal.pone.0294146)
Supplement: S3 Fig — Proliferation indices of the cells listed on the right were assessed by MTT assay. Cell numbers at 24–120 h after cell seeding were evaluated every 24 h and are indicated relative to those at 24 h after cell seeding. Data represent the mean and SEM values from three independent experiments. Quadruplicate samples were analyzed in each experiment. (PDF) [file pone.0294146.s003.pdf]

S3 Fig

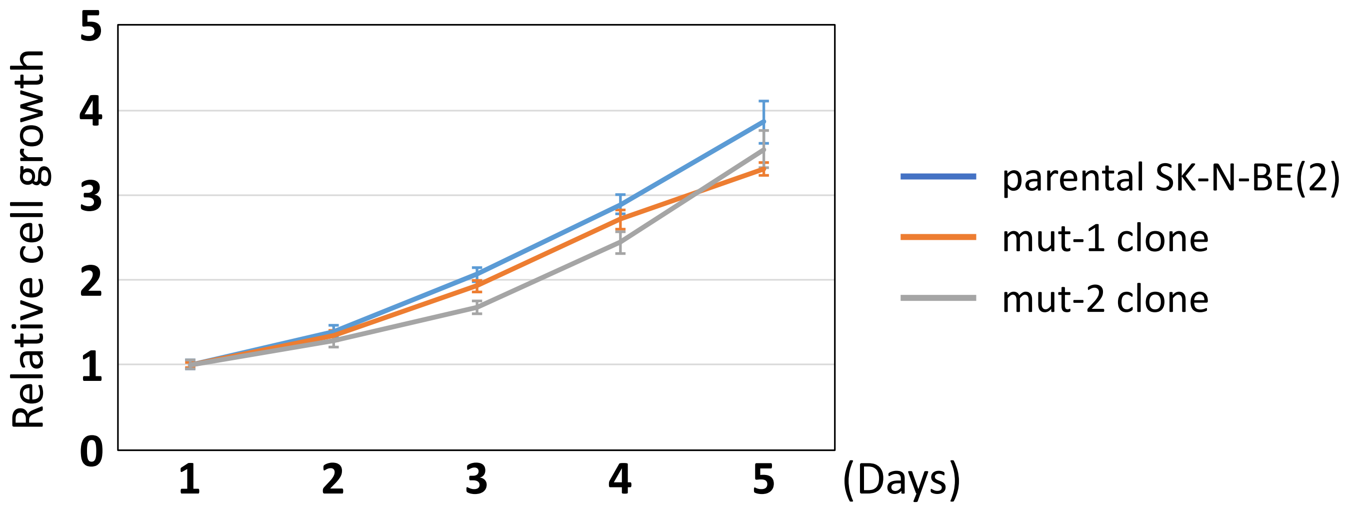

S3 Fig. The mut-1 and mut-2 reporter clones and their parental SK-N-BE(2) cell line exhibit similar proliferation indices.

Proliferation indices of the cells listed on the right were assessed by MTT assay. Cell numbers at 24–120 h after cell seeding were evaluated every 24 h and are indicated relative to those at 24 h after cell seeding. Data represent the mean and SEM values from three independent experiments. Quadruplicate samples were analyzed in each experiment.
