# Supplementary material for: Flow cytometry-based quantification of genome editing efficiency in human cell lines using the L1CAM gene
Source: PLoS One. 2023 Nov 9;18(11):e0294146. doi: 10.1371/journal.pone.0294146 (PMC10635454; doi:10.1371/journal.pone.0294146)
Supplement: S4 Fig — SK-N-BE(2) cells were transfected with a Cas9 nuclease targeted to L1CAM (ex26-1), and the L1CAM protein on the surface of transfected cells was labeled with Alexa Fluor 488. Cells were then FCM-sorted to isolate Alexa Fluor 488-positive and -negative cells. Next, a genomic region spanning the edited site at the L1CAM intron 25–exon 26 boundary was PCR-amplified using gDNAs extracted from the Alexa Fluor 488-positive and -negative cells as templates. Lastly, the amplified PCR products were cloned into a plasmid, and multiple plasmids containing the PCR products as inserts were isolated and sequenced. (A) DNA sequences of PCR products amplified from Alexa Fluor 488-positive cells. Sequences are shown in alignment with a wild-type control derived from parental SK-N-BE(2) cells displayed at the top. A red letter indicates an inserted nucleotide. (B) Representative sequencing chromatogram obtained in the analysis shown in (A). (C) DNA sequences of PCR products amplified from Alexa Fluor 488-negative cells displayed in a manner similar to (A). A green letter indicates a substituted nucleotide. (D) Two representative sequencing chromatograms obtained in the analysis shown in (C). (E) L1CAM genotypes in Alexa Fluor 488-positive and -negative cells determined based on the experimental results shown in (A)–(D). In (A)–(D), the vertical dotted lines in red indicate a genomic site cleaved by ex26-1. WT, wild-type; ins, insertion; del, deletion; subst, substitution. (PDF) [file pone.0294146.s004.pdf]

S4 Fig-1

A

```
Parent     ggcttgggcggcagcacggggggctggtgtctcaccctcagGCCGCGTGAGGCTCCCTCCTGCTGGCTTCGCCACTGAGG
Clone 1    ggcttgggcggcagcacggggggctggtgtctcaccctcagGCCGCGTGAGGCTCCCTCCTGCTGGCTTCGCCACTGAGG    WT
Clone 2    ggcttgggcggcagcacggggggctggtgtctcaccctcagGCCGCGTGAGGCTCCCTCCTGCTGGCTTCGCCACTGAGG    WT
Clone 3    ggcttgggcggcagcacggggggctggtgtctcaccctcagGCCGCGTGAGGCTCCCTCCTGCTGGCTTCGCCACTGAGG    WT
Clone 4    ggcttgggcggcagcacggggggctggtgtctcaccctcagGCCGCGTGAGGCTCCCTCCTGCTGGCTTCGCCACTGAGG    WT
Clone 5    ggcttgggcggcagcacggggggctggtgtctcaccctcaggGCCGCGTGAGGCTCCCTCCTGCTGGCTTCGCCACTGAGG   1 bp ins
Clone 6    ggcttgggcggcagcacggggggctggtgtctcaccctcagGCCGCGTGAGGCTCCCTCCTGCTGGCTTCGCCACTGAGG    WT
Clone 7    ggcttgggcggcagcacggggggctggtgtctcaccctcagGCCGCGTGAGGCTCCCTCCTGCTGGCTTCGCCACTGAGG    WT
Clone 8    ggcttgggcggcagcacggggggctggtgtctcaccctcagGCCGCGTGAGGCTCCCTCCTGCTGGCTTCGCCACTGAGG    WT
Clone 9    ggcttgggcggcagcacggggggctggtgtctcaccctcagGCCGCGTGAGGCTCCCTCCTGCTGGCTTCGCCACTGAGG    WT
Clone 10   ggcttgggcggcagcacggggggctggtgtctcaccctcagGCCGCGTGAGGCTCCCTCCTGCTGGCTTCGCCACTGAGG    WT
Clone 11   ggcttgggcggcagcacggggggctggtgtctcaccctcagGCCGCGTGAGGCTCCCTCCTGCTGGCTTCGCCACTGAGG    WT
Clone 12   ggcttgggcggcagcacggggggctggtgtctcaccctcagGCCGCGTGAGGCTCCCTCCTGCTGGCTTCGCCACTGAGG    WT
Clone 13   ggcttgggcggcagcacggggggctggtgtctcaccctcagGCCGCGTGAGGCTCCCTCCTGCTGGCTTCGCCACTGAGG    WT
Clone 14   ggcttgggcggcagcacggggggctggtgtctcaccctcagGCCGCGTGAGGCTCCCTCCTGCTGGCTTCGCCACTGAGG    WT
```

B

Clone 1

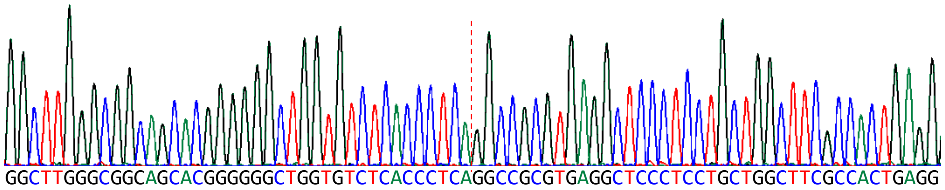

C

```
Parent     ggcttgggcggcagcacggggggctggtgtctcaccctcagGCCGCGTGAGGCTCCCTCCTGCTGGCTTCGCCACTGAGG
Clone 1    ggcttgggcggcagcacgggg-------------------gGCCGCGTGAGGCTCCCTCCTGCTGGCTTCGCCACTGAGG            19 bp del
Clone 2    ggcttgggcggcagcacggggggct----------------GCCGCGTGAGGCTCCCTCCTGCTGGCTTCGCCACTGAGG            16 bp del
Clone 3    ggcttgggcggcagcacggggggctggtgtctcaccctcaggGCCGCGTGAGGCTCCCTCCTGCTGGCTTCGCCACTGAGG            1 bp ins
Clone 4    ggcttgggcggcagcacggggggctggtgtctcaccctcaggGCCGCGTGAGGCTCCCTCCTGCTGGCTTCGCCACTGAGG            1 bp ins
Clone 5    ggcttgggcggcagcacggggggctggtgtctcaccctca-GCCGCGTGAGGCTCCCTCCTGCTGGCTTCGCCACTGAGG            1 bp del
Clone 6    ggcttgggc------------------------------a----GCGTGAGGCTCCCTCCTGCTGGCTTCGCCACTGAGG   35 bp del + 1 bp ins
Clone 7    ggcttgggcggcagcacggggggctggtgtctca------gGCCGCGTGAGGCTCCCTCCTGCTGGCTTCGCCACTGAGG             6 bp del
Clone 8    ggcttgggcggcagcactgggggctggtgtctcaccctcaggGCCGCGTGAGGCTCCCTCCTGCTGGCTTCGCCACTGAGG  1 bp ins + 1 bp subst
Clone 9    ggcttgggcggcagcacggggggctggtgt----------------CTGAGGCTCCCTCCTGCTGGCTTCGCCACTGAGG            16 bp del
Clone 10   ggcttgggcggcagcacggggggctggtgtctcaccctcaggGCCGCGTGAGGCTCCCTCCTGCTGGCTTCGCCACTGAGG            1 bp ins
Clone 11   ggcttgggcggcagcacggggggctggtgtctcaccctcaggGCCGCGTGAGGCTCCCTCCTGCTGGCTTCGCCACTGAGG            1 bp ins
Clone 12   ggcttgggcggcagcacggggggct----------------GCCGCGTGAGGCTCCCTCCTGCTGGCTTCGCCACTGAGG            16 bp del
Clone 13   ggcttgggcggcagcacggggggctggtgtctcaccctcagG-CGCGTGAGGCTCCCTCCTGCTGGCTTCGCCACTGAGG            1 bp del
Clone 14   ggcttgggcggcagcacggggggctgg---------------------------------TGCTGGCTTCGCCACTGAGG            33 bp del
Clone 15   ggcttgggcggcagcacggggggctggtgtctcaccctca-GCCGCGTGAGGCTCCCTCCTGCTGGCTTCGCCACTGAGG            1 bp del
Clone 16   ggcttgggcggcagcacggggggctggtgt----------------------CTCCCTCCTGCTGGCTTCGCCACTGAGG            22 bp del
Clone 17   ggcttgggcggcagcacggggggctggtgtctcaccctcaggGCCGCGTGAGGCTCCCTCCTGCTGGCTTCGCCACTGAGG            1 bp ins
Clone 18   ggcttgggcggcagcacggggggct----------------GCCGCGTGAGGCTCCCTCCTGCTGGCTTCGCCACTGAGG            16 bp del
Clone 19   ggcttgggcggcagcacggggggct-------------------------------------------TCGCCACTGAGG            43 bp del
Clone 20   ggcttgggcggcagcacggggggctggtgtctc-------gGCCGCGTGAGGCTCCCTCCTGCTGGCTTCGCCACTGAGG             7 bp del
Clone 21   ggcttgggcggcagcacggggggct----------------GCCGCGTGAGGCTCCCTCCTGCTGGCTTCGCCACTGAGG            16 bp del
Clone 22   ggcttgggcggcagcacggggggct----------------GCCGCGTGAGGCTCCCTCCTGCTGGCTTCGCCACTGAGG            16 bp del
Clone 23   ggcttgggcggcagcacggg-------------------a----GGCGTGAGGCTCCCTCCTGCTGGCTTCGCCACTGAGG  24 bp del + 1 bp ins
Clone 24   ggcttgggcggcagcacggggggctggtgtctcaccctca--CCGCGTGAGGCTCCCTCCTGCTGGCTTCGCCACTGAGG             2 bp del
Clone 25   ggcttgggcggcagcacggggggct----------------GCCGCGTGAGGCTCCCTCCTGCTGGCTTCGCCACTGAGG            16 bp del
Clone 26   ggcttgggcggcagcacggggggctggtgtctcaccctcag-----------------CCTGCTGGCTTCGCCACTGAGG            17 bp del
Clone 27   ggcttgggcggcagcacggggggctggtgtctcaccctcaggGCCGCGTGAGGCTCCCTCCTGCTGGCTTCGCCACTGAGG            1 bp ins
Clone 28   ggcttgggcggcagcacggggggctggtgtctcaccctcaggGCCGCGTGAGGCTCCCTCCTGCTGGCTTCGCCACTGAGG            1 bp ins
Clone 29   ggcttgggcggcagcacggggggctggtgtctcac-----gGCCGCGTGAGGCTCCCTCCTGCTGGCTTCGCCACTGAGG             5 bp del
Clone 30   ggcttgggcggcagcacggggggctggtgtctcaccctcaggGCCGCGTGAGGCTCCCTCCTGCTGGCTTCGCCACTGAGG            1 bp ins
```

D

Clone 1

Clone 3

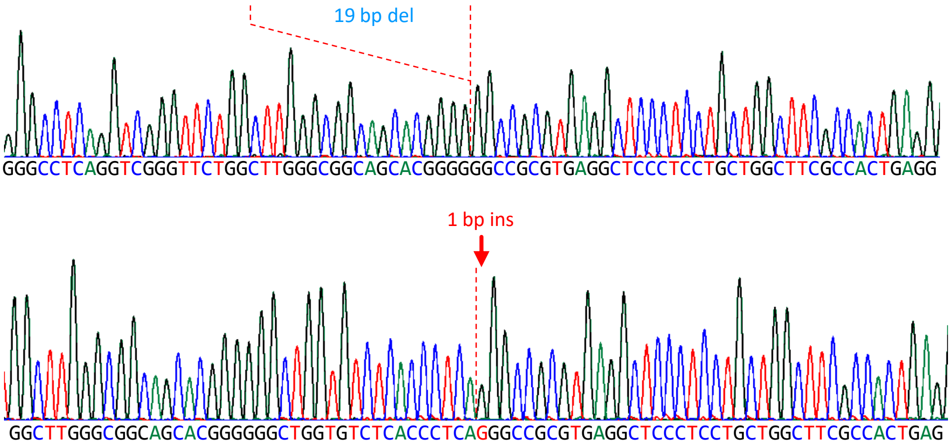

(S4 Fig continued on the next page)

S4 Fig-2

E

	L1CAM intron 25 – exon 26 boundary		
	Wild-type	Indels	
Alexa Fluor 488-positive	93 %	7 %	(n = 14)
Alexa Fluor 488-negative	0 %	100 %	(n = 30)

S4 Fig. Alexa Fluor 488 negativity in the *L1CAM* assay represents *L1CAM* disruption by indels.
SK-N-BE(2) cells were transfected with a Cas9 nuclease targeted to *L1CAM* (ex26-1), and the L1CAM protein on the surface of transfected cells was labeled with Alexa Fluor 488. Cells were then FCM-sorted to isolate Alexa Fluor 488-positive and -negative cells. Next, a genomic region spanning the edited site at the *L1CAM* intron 25–exon 26 boundary was PCR-amplified using gDNAs extracted from the Alexa Fluor 488-positive and -negative cells as templates. Lastly, the amplified PCR products were cloned into a plasmid, and multiple plasmids containing the PCR products as inserts were isolated and sequenced.
(A) DNA sequences of PCR products amplified from Alexa Fluor 488-positive cells. Sequences are shown in alignment with a wild-type control derived from parental SK-N-BE(2) cells displayed at the top. A red letter indicates an inserted nucleotide.
(B) Representative sequencing chromatogram obtained in the analysis shown in (A).
(C) DNA sequences of PCR products amplified from Alexa Fluor 488-negative cells displayed in a manner similar to (A). A green letter indicates a substituted nucleotide.
(D) Two representative sequencing chromatograms obtained in the analysis shown in (C).
(E) *L1CAM* genotypes in Alexa Fluor 488-positive and -negative cells determined based on the experimental results shown in (A)–(D). In (A)–(D), the vertical dotted lines in red indicate a genomic site cleaved by ex26-1. WT, wild-type; ins, insertion; del, deletion; subst, substitution.
