# Supplementary material for: Flow cytometry-based quantification of genome editing efficiency in human cell lines using the L1CAM gene
Source: PLoS One. 2023 Nov 9;18(11):e0294146. doi: 10.1371/journal.pone.0294146 (PMC10635454; doi:10.1371/journal.pone.0294146)
Supplement: S5 Fig — (A) L1CAM exons 2–4 were amplified using cDNA from SK-N-BE(2) as a template, and the resultant bulk PCR product was Sanger sequenced. Top, a scheme of L1CAM exons 1–5 associated with primers used for PCR (green arrows). Bottom, sequencing chromatograms obtained using forward (F) and reverse (R) sequencing primes. Annotations “mixed” indicate that an intermixture of multiple sequences initiates immediately after the terminus of L1CAM exon 2 (F) and exon 4 (R), consistent with the alternative splicing of exon 3. (B) The bulk PCR product obtained in (A) was fractionated on an agarose gel as shown on the left. Two distinct PCR products of approximately 300-bp in size were isolated from the gel and Sanger sequenced using a forward sequencing primer. Shown on the right are two chromatograms, one exhibiting a sequence of L1CAM exons 2–4 and the other revealing joined sequences of L1CAM exons 2 and 4. Primers used for PCR amplification and Sanger sequencing are listed in S3 Table. (PDF) [file pone.0294146.s005.pdf]

S5 Fig

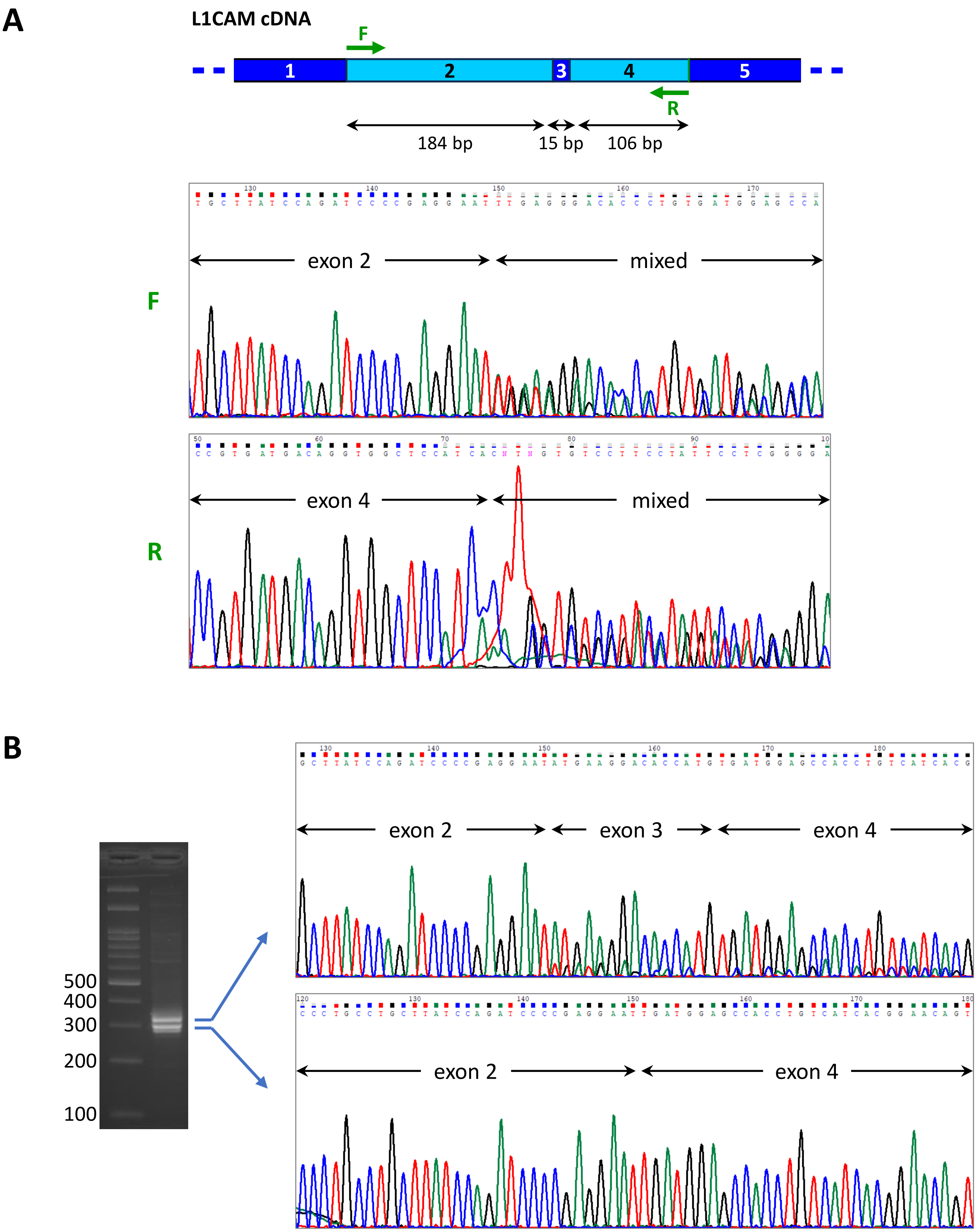

S5 Fig. ***L1CAM* exon 3 is an alternative exon.**
(A) *L1CAM* exons 2–4 were amplified using cDNA from SK-N-BE(2) as a template, and the resultant bulk PCR product was Sanger sequenced. Top, a scheme of *L1CAM* exons 1–5 associated with primers used for PCR (green arrows). Bottom, sequencing chromatograms obtained using forward (F) and reverse (R) sequencing primes. Annotations "mixed" indicate that an intermixture of multiple sequences initiates immediately after the terminus of *L1CAM* exon 2 (F) and exon 4 (R), consistent with the alternative splicing of exon 3.

(B) The bulk PCR product obtained in (A) was fractionated on an agarose gel as shown on the left. Two distinct PCR products of approximately 300-bp in size were isolated from the gel and Sanger sequenced using a forward sequencing primer. Shown on the right are two chromatograms, one exhibiting a sequence of *L1CAM* exons 2–4 and the other revealing joined sequences of *L1CAM* exons 2 and 4. Primers used for PCR amplification and Sanger sequencing are listed in S3 Table.
