# Supplementary material for: Flow cytometry-based quantification of genome editing efficiency in human cell lines using the L1CAM gene
Source: PLoS One. 2023 Nov 9;18(11):e0294146. doi: 10.1371/journal.pone.0294146 (PMC10635454; doi:10.1371/journal.pone.0294146)
Supplement: S6 Fig — Target sequences of Cas9 nucleases (A and B, brown) and Cas9 nickases (#1–#6, green) are color-shaded, with darker shading on the neighboring PAMs. Brown and green numbers are arbitrary values showing the relative positions of cleavage by Cas9 nucleases and nickases, respectively. Nucleotides indicated by bold letters with pink and blue shading represent mut-1 and mut-2 truncating mutations in the reporter clones and their corresponding wild-type sequences in Donor-L1CAM, respectively. Uppercase and lowercase letters in DNA sequences indicate exonic and intronic sequences, respectively. (PDF) [file pone.0294146.s006.pdf]

S6 Fig

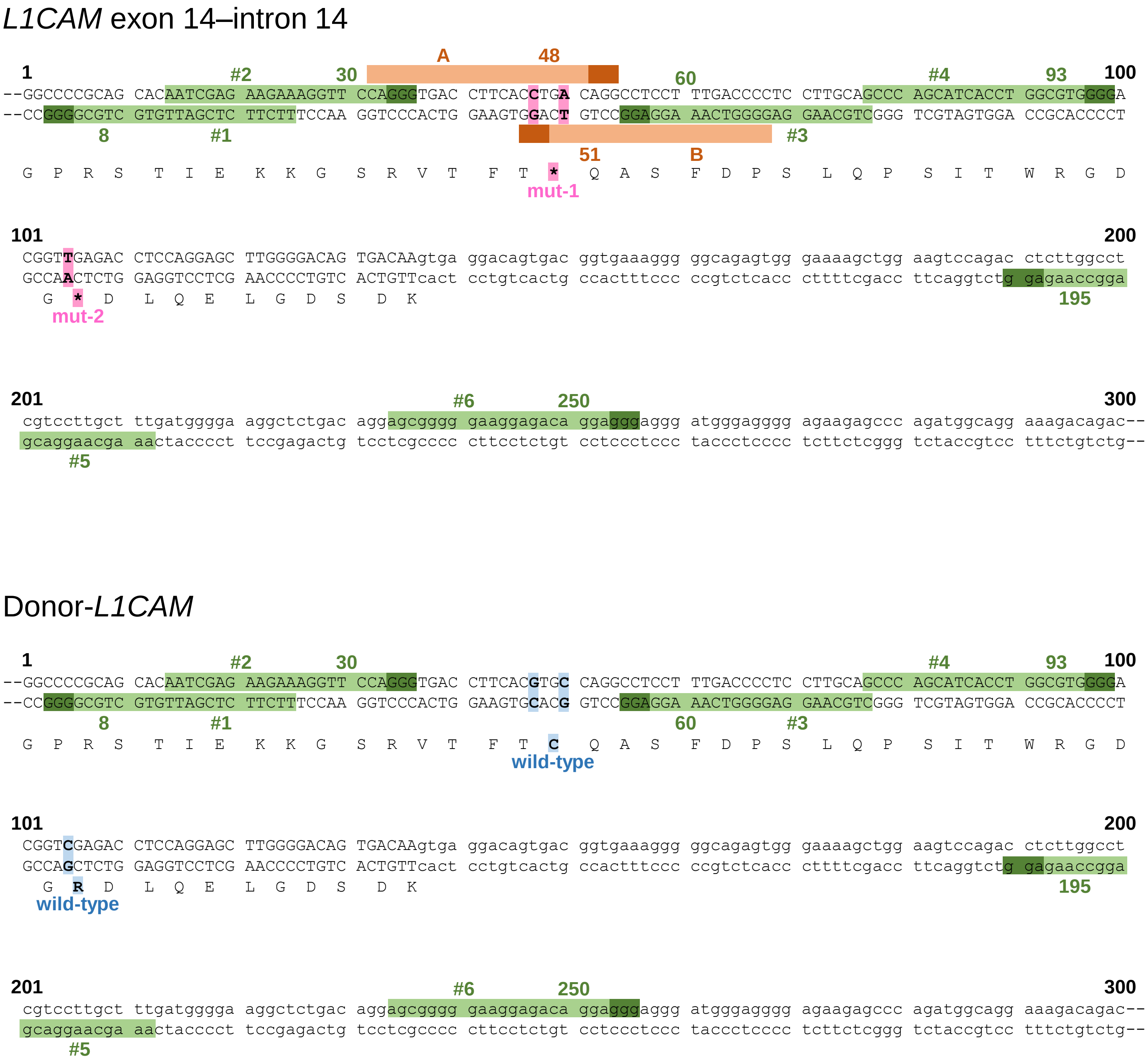

S6 Fig. DNA sequences surrounding the mutated genomic sites at *L1CAM* exon 14 in the SK-N-BE(2)-derived mut-1 and mut-2 reporter clones (top) and the corresponding wild-type sequence in the Donor-*L1CAM* plasmid (bottom).
Target sequences of Cas9 nucleases (A and B, brown) and Cas9 nickases (#1–#6, green) are color-shaded, with darker shading on the neighboring PAMs. Brown and green numbers are arbitrary values showing the relative positions of cleavage by Cas9 nucleases and nickases, respectively. Nucleotides indicated by bold letters with pink and blue shading represent mut-1 and mut-2 truncating mutations in the reporter clones and their corresponding wild-type sequences in Donor-*L1CAM*, respectively. Uppercase and lowercase letters in DNA sequences indicate exonic and intronic sequences, respectively.
