# Supplementary material for: Flow cytometry-based quantification of genome editing efficiency in human cell lines using the L1CAM gene
Source: PLoS One. 2023 Nov 9;18(11):e0294146. doi: 10.1371/journal.pone.0294146 (PMC10635454; doi:10.1371/journal.pone.0294146)
Supplement: S7 Fig — Letters with pink and blue shading indicate mutated and wild-type sequences, respectively. (PDF) [file pone.0294146.s007.pdf]

S7 Fig

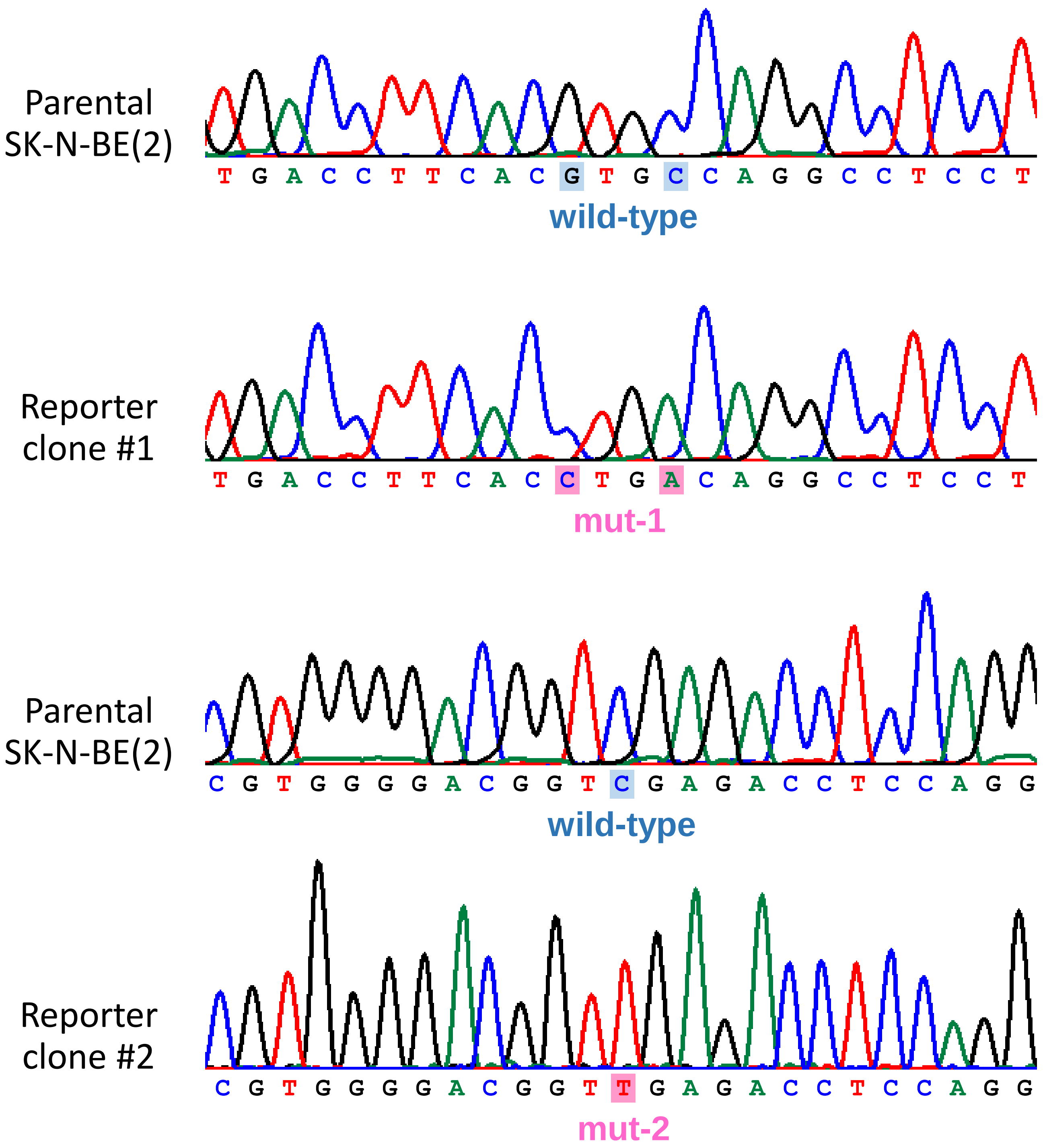

S7 Fig. Sequence chromatograms showing mutated genomic sites in the mut-1 and mut-2 reporter clones and the corresponding genomic sites in the parental SK-N-BE(2) cell line.
Letters with pink and blue shading indicate mutated and wild-type sequences, respectively.
