# Supplementary material for: Flow cytometry-based quantification of genome editing efficiency in human cell lines using the L1CAM gene
Source: PLoS One. 2023 Nov 9;18(11):e0294146. doi: 10.1371/journal.pone.0294146 (PMC10635454; doi:10.1371/journal.pone.0294146)
Supplement: S8 Fig — The SK-N-BE(2)-derived mut-2 reporter clone was transfected with sgRNA #4 and #6 coupled with Cas9 (H840A) and Donor-L1CAM to correct the L1CAM mutation via TPN. The L1CAM protein on the surface of transfected cells was labeled with Alexa Fluor 488, and cells were subjected to FCM-based sorting to isolate Alexa Fluor 488-positive and -negative populations. PCR was then performed to amplify a genomic region spanning the mut-2 site within L1CAM exon 14 in the Alexa Fluor 488-positive and -negative populations. The amplified PCR products were cloned into a plasmid, and multiple plasmids containing the PCR products as inserts were isolated and sequenced. (A) DNA sequences of PCR products amplified from Alexa Fluor 488-positive cells. Sequences are shown in alignment with a wild-type control derived from parental SK-N-BE(2) cells displayed at the top. Arbitrary numbers placed above the aligned sequences indicate the relative positions of nucleotides. Blue shading indicates a wild-type sequence resulting from mut-2 reversion. Green letters indicate substituted nucleotides. (B) Representative sequencing chromatogram obtained in the analysis shown in (A). (C) DNA sequences of PCR products amplified from Alexa Fluor 488-negative cells displayed in a manner similar to (A). Red shading indicates the mut-2 nonsense mutation. (D) Representative sequencing chromatogram obtained in the analysis shown in (C). (E) L1CAM genotypes in Alexa Fluor 488-positive and -negative cells determined based on the experimental results shown in (A)–(D). 1-bp substitutions shown in (A) and (C), probably introduced during genome editing or by PCR errors, are not considered in genotyping L1CAM, because they are located within an intronic sequence distant from the exon–intron boundary. In (A)–(D), the vertical dotted lines in red indicate a genomic site nicked by Cas9 (H840A) coupled with sgRNA #4 or #6. WT, wild-type; mut, mutant. (PDF) [file pone.0294146.s008.pdf]

S8 Fig-1

A

```
           1                                                                              80
Parent     GCCTCCTTTGACCCCTCCTTGCAGCCCAGCATCACCTGGCGTGGGGACGGTCGAGACCTCCAGGAGCTTGGGGACAGTGA
Clone 1    GCCTCCTTTGACCCCTCCTTGCAGCCCAGCATCACCTGGCGTGGGGACGGTCGAGACCTCCAGGAGCTTGGGGACAGTGA         WT
Clone 2    GCCTCCTTTGACCCCTCCTTGCAGCCCAGCATCACCTGGCGTGGGGACGGTCGAGACCTCCAGGAGCTTGGGGACAGTGA         WT
Clone 3    GCCTCCTTTGACCCCTCCTTGCAGCCCAGCATCACCTGGCGTGGGGACGGTCGAGACCTCCAGGAGCTTGGGGACAGTGA         WT
Clone 4    GCCTCCTTTGACCCCTCCTTGCAGCCCAGCATCACCTGGCGTGGGGACGGTCGAGACCTCCAGGAGCTTGGGGACAGTGA 1 bp subst
Clone 5    GCCTCCTTTGACCCCTCCTTGCAGCCCAGCATCACCTGGCGTGGGGACGGTCGAGACCTCCAGGAGCTTGGGGACAGTGA         WT
Clone 6    GCCTCCTTTGACCCCTCCTTGCAGCCCAGCATCACCTGGCGTGGGGACGGTCGAGACCTCCAGGAGCTTGGGGACAGTGA         WT
Clone 7    GCCTCCTTTGACCCCTCCTTGCAGCCCAGCATCACCTGGCGTGGGGACGGTCGAGACCTCCAGGAGCTTGGGGACAGTGA         WT
Clone 8    GCCTCCTTTGACCCCTCCTTGCAGCCCAGCATCACCTGGCGTGGGGACGGTCGAGACCTCCAGGAGCTTGGGGACAGTGA         WT
Clone 9    GCCTCCTTTGACCCCTCCTTGCAGCCCAGCATCACCTGGCGTGGGGACGGTCGAGACCTCCAGGAGCTTGGGGACAGTGA         WT
Clone 10   GCCTCCTTTGACCCCTCCTTGCAGCCCAGCATCACCTGGCGTGGGGACGGTCGAGACCTCCAGGAGCTTGGGGACAGTGA         WT
Clone 11   GCCTCCTTTGACCCCTCCTTGCAGCCCAGCATCACCTGGCGTGGGGACGGTCGAGACCTCCAGGAGCTTGGGGACAGTGA         WT
Clone 12   GCCTCCTTTGACCCCTCCTTGCAGCCCAGCATCACCTGGCGTGGGGACGGTCGAGACCTCCAGGAGCTTGGGGACAGTGA         WT
Clone 13   GCCTCCTTTGACCCCTCCTTGCAGCCCAGCATCACCTGGCGTGGGGACGGTCGAGACCTCCAGGAGCTTGGGGACAGTGA 1 bp subst
Clone 14   GCCTCCTTTGACCCCTCCTTGCAGCCCAGCATCACCTGGCGTGGGGACGGTCGAGACCTCCAGGAGCTTGGGGACAGTGA         WT

           81                                                                            160
Parent     CAAgtgaggacagtgacggtgaaagggggcagagtgggaaaagctggaagtccagacctcttggcctcgtccttgctttg
Clone 1    CAAgtgaggacagtgacggtgaaagggggcagagtgggaaaagctggaagtccagacctcttggcctcgtccttgctttg         WT
Clone 2    CAAgtgaggacagtgacggtgaaagggggcagagtgggaaaagctggaagtccagacctcttggcctcgtccttgctttg         WT
Clone 3    CAAgtgaggacagtgacggtgaaagggggcagagtgggaaaagctggaagtccagacctcttggcctcgtccttgctttg         WT
Clone 4    CAAgtgaggacagtgacggtgaaagggggtagagtgggaaaagctggaagtccagacctcttggcctcgtccttgctttg 1 bp subst
Clone 5    CAAgtgaggacagtgacggtgaaagggggcagagtgggaaaagctggaagtccagacctcttggcctcgtccttgctttg         WT
Clone 6    CAAgtgaggacagtgacggtgaaagggggcagagtgggaaaagctggaagtccagacctcttggcctcgtccttgctttg         WT
Clone 7    CAAgtgaggacagtgacggtgaaagggggcagagtgggaaaagctggaagtccagacctcttggcctcgtccttgctttg         WT
Clone 8    CAAgtgaggacagtgacggtgaaagggggcagagtgggaaaagctggaagtccagacctcttggcctcgtccttgctttg         WT
Clone 9    CAAgtgaggacagtgacggtgaaagggggcagagtgggaaaagctggaagtccagacctcttggcctcgtccttgctttg         WT
Clone 10   CAAgtgaggacagtgacggtgaaagggggcagagtgggaaaagctggaagtccagacctcttggcctcgtccttgctttg         WT
Clone 11   CAAgtgaggacagtgacggtgaaagggggcagagtgggaaaagctggaagtccagacctcttggcctcgtccttgctttg         WT
Clone 12   CAAgtgaggacagtgacggtgaaagggggcagagtgggaaaagctggaagtccagacctcttggcctcgtccttgctttg         WT
Clone 13   CAAgtgaggacagtgacggtgaaagggggcagagtgggaaaagctggaagtccagacctcttggcctcgtccttgctttg 1 bp subst
Clone 14   CAAgtgaggacagtgacggtgaaagggggcagagtgggaaaagctggaagtccagacctcttggcctcgtccttgctttg         WT

           161                                                                           240
Parent     atggggaaggctctgacaggagcgggggaaggagacaggagggagggatgggaggggagaagagcccagatggcaggaaa
Clone 1    atggggaaggctctgacaggagcgggggaaggagacaggagggagggatgggaggggagaagagcccagatggcaggaaa         WT
Clone 2    atggggaaggctctgacaggagcgggggaaggagacaggagggagggatgggaggggagaagagcccagatggcaggaaa         WT
Clone 3    atggggaaggctctgacaggagcgggggaaggagacaggagggagggatgggaggggagaagagcccagatggcaggaaa         WT
Clone 4    atggggaaggctctgacaggagcgggggaaggagacaggagggagggatgggaggggagaagagcccagatggcaggaaa 1 bp subst
Clone 5    atggggaaggctctgacaggagcgggggaaggagacaggagggagggatgggaggggagaagagcccagatggcaggaaa         WT
Clone 6    atggggaaggctctgacaggagcgggggaaggagacaggagggagggatgggaggggagaagagcccagatggcaggaaa         WT
Clone 7    atggggaaggctctgacaggagcgggggaaggagacaggagggagggatgggaggggagaagagcccagatggcaggaaa         WT
Clone 8    atggggaaggctctgacaggagcgggggaaggagacaggagggagggatgggaggggagaagagcccagatggcaggaaa         WT
Clone 9    atggggaaggctctgacaggagcgggggaaggagacaggagggagggatgggaggggagaagagcccagatggcaggaaa         WT
Clone 10   atggggaaggctctgacaggagcgggggaaggagacaggagggagggatgggaggggagaagagcccagatggcaggaaa         WT
Clone 11   atggggaaggctctgacaggagcgggggaaggagacaggagggagggatgggaggggagaagagcccagatggcaggaaa         WT
Clone 12   atggggaaggctctgacaggagcgggggaaggagacaggagggagggatgggaggggagaagagcccagatggcaggaaa         WT
Clone 13   atggggaaggctctgacaggagcgggggaaggagacaggaggtagggatgggaggggagaagagcccagatggcaggaaa 1 bp subst
Clone 14   atggggaaggctctgacaggagcgggggaaggagacaggagggagggatgggaggggagaagagcccagatggcaggaaa         WT
```

B

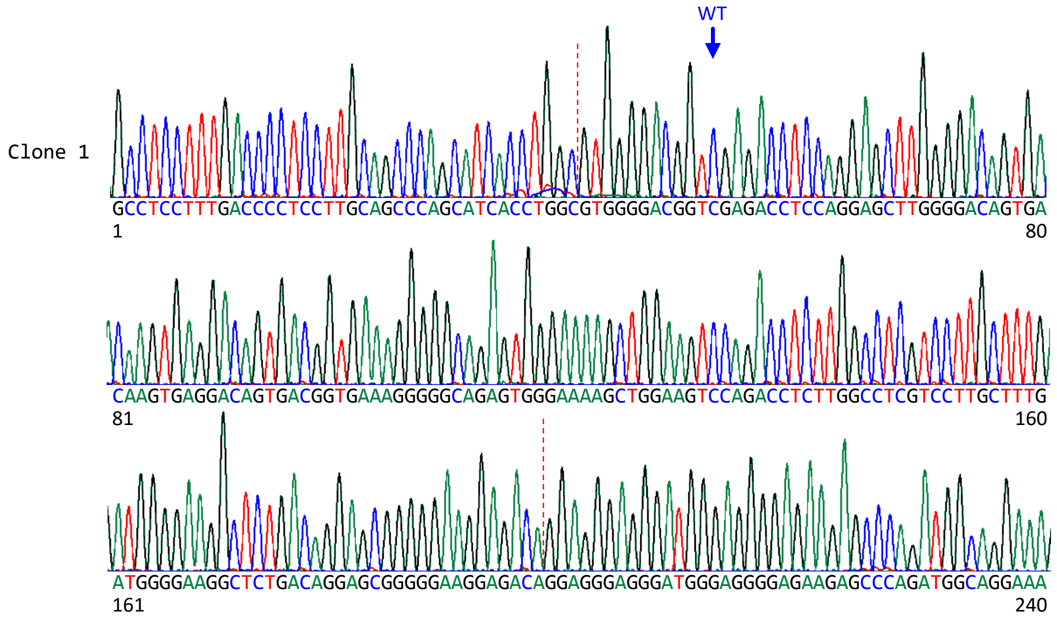

(S8 Fig continued on the next page)

S8 Fig-2

C

```
             1                                                                              80
Parent       GCCTCCTTTGACCCCTCCTTGCAGCCCAGCATCACCTGGCGTGGGGACGGTCGAGACCTCCAGGAGCTTGGGGACAGTGA
Clone 1      GCCTCCTTTGACCCCTCCTTGCAGCCCAGCATCACCTGGCGTGGGGACGGTTGAGACCTCCAGGAGCTTGGGGACAGTGA                  mut
Clone 2      GCCTCCTTTGACCCCTCCTTGCAGCCCAGCATCACCTGGCGTGGGGACGGTTGAGACCTCCAGGAGCTTGGGGACAGTGA                  mut
Clone 3      GCCTCCTTTGACCCCTCCTTGCAGCCCAGCATCACCTGGCGTGGGGACGGTTGAGACCTCCAGGAGCTTGGGGACAGTGA                  mut
Clone 4      GCCTCCTTTGACCCCTCCTTGCAGCCCAGCATCACCTGGCGTGGGGACGGTTGAGACCTCCAGGAGCTTGGGGACAGTGA                  mut
Clone 5      GCCTCCTTTGACCCCTCCTTGCAGCCCAGCATCACCTGGCGTGGGGACGGTTGAGACCTCCAGGAGCTTGGGGACAGTGA     mut + 1 bp subst
Clone 6      GCCTCCTTTGACCCCTCCTTGCAGCCCAGCATCACCTGGCGTGGGGACGGTTGAGACCTCCAGGAGCTTGGGGACAGTGA                  mut
Clone 7      GCCTCCTTTGACCCCTCCTTGCAGCCCAGCATCACCTGGCGTGGGGACGGTTGAGACCTCCAGGAGCTTGGGGACAGTGA                  mut
Clone 8      GCCTCCTTTGACCCCTCCTTGCAGCCCAGCATCACCTGGCGTGGGGACGGTTGAGACCTCCAGGAGCTTGGGGACAGTGA                  mut
Clone 9      GCCTCCTTTGACCCCTCCTTGCAGCCCAGCATCACCTGGCGTGGGGACGGTTGAGACCTCCAGGAGCTTGGGGACAGTGA                  mut
Clone 10     GCCTCCTTTGACCCCTCCTTGCAGCCCAGCATCACCTGGCGTGGGGACGGTTGAGACCTCCAGGAGCTTGGGGACAGTGA     mut + 1 bp subst
Clone 11     GCCTCCTTTGACCCCTCCTTGCAGCCCAGCATCACCTGGCGTGGGGACGGTTGAGACCTCCAGGAGCTTGGGGACAGTGA                  mut
Clone 12     GCCTCCTTTGACCCCTCCTTGCAGCCCAGCATCACCTGGCGTGGGGACGGTTGAGACCTCCAGGAGCTTGGGGACAGTGA                  mut
Clone 13     GCCTCCTTTGACCCCTCCTTGCAGCCCAGCATCACCTGGCGTGGGGACGGTTGAGACCTCCAGGAGCTTGGGGACAGTGA                  mut
Clone 14     GCCTCCTTTGACCCCTCCTTGCAGCCCAGCATCACCTGGCGTGGGGACGGTTGAGACCTCCAGGAGCTTGGGGACAGTGA                  mut

             81                                                                            160
Parent       CAAgtgaggacagtgacggtgaaagggggcagagtgggaaaagctggaagtccagacctcttggcctcgtccttgctttg
Clone 1      CAAgtgaggacagtgacggtgaaagggggcagagtgggaaaagctggaagtccagacctcttggcctcgtccttgctttg                  mut
Clone 2      CAAgtgaggacagtgacggtgaaagggggcagagtgggaaaagctggaagtccagacctcttggcctcgtccttgctttg                  mut
Clone 3      CAAgtgaggacagtgacggtgaaagggggcagagtgggaaaagctggaagtccagacctcttggcctcgtccttgctttg                  mut
Clone 4      CAAgtgaggacagtgacggtgaaagggggcagagtgggaaaagctggaagtccagacctcttggcctcgtccttgctttg                  mut
Clone 5      CAAgtgaggacagtgacggtgaaagggggcagagtgagaaaagctggaagtccagacctcttggcctcgtccttgctttg     mut + 1 bp subst
Clone 6      CAAgtgaggacagtgacggtgaaagggggcagagtgggaaaagctggaagtccagacctcttggcctcgtccttgctttg                  mut
Clone 7      CAAgtgaggacagtgacggtgaaagggggcagagtgggaaaagctggaagtccagacctcttggcctcgtccttgctttg                  mut
Clone 8      CAAgtgaggacagtgacggtgaaagggggcagagtgggaaaagctggaagtccagacctcttggcctcgtccttgctttg                  mut
Clone 9      CAAgtgaggacagtgacggtgaaagggggcagagtgggaaaagctggaagtccagacctcttggcctcgtccttgctttg                  mut
Clone 10     CAAgtgaggacagtgacggtgaaagggggcagagtgggaaaagctggaagtccagacctcttggcctcgtccttgctttg     mut + 1 bp subst
Clone 11     CAAgtgaggacagtgacggtgaaagggggcagagtgggaaaagctggaagtccagacctcttggcctcgtccttgctttg                  mut
Clone 12     CAAgtgaggacagtgacggtgaaagggggcagagtgggaaaagctggaagtccagacctcttggcctcgtccttgctttg                  mut
Clone 13     CAAgtgaggacagtgacggtgaaagggggcagagtgggaaaagctggaagtccagacctcttggcctcgtccttgctttg                  mut
Clone 14     CAAgtgaggacagtgacggtgaaagggggcagagtgggaaaagctggaagtccagacctcttggcctcgtccttgctttg                  mut

             161                                                                           240
Parent       atggggaaggctctgacaggagcgggggaaggagacaggagggagggatgggaggggagaagagcccagatggcaggaaa
Clone 1      atggggaaggctctgacaggagcgggggaaggagacaggagggagggatgggaggggagaagagcccagatggcaggaaa                  mut
Clone 2      atggggaaggctctgacaggagcgggggaaggagacaggagggagggatgggaggggagaagagcccagatggcaggaaa                  mut
Clone 3      atggggaaggctctgacaggagcgggggaaggagacaggagggagggatgggaggggagaagagcccagatggcaggaaa                  mut
Clone 4      atggggaaggctctgacaggagcgggggaaggagacaggagggagggatgggaggggagaagagcccagatggcaggaaa                  mut
Clone 5      atggggaaggctctgacaggagcgggggaaggagacaggagggagggatgggaggggagaagagcccagatggcaggaaa     mut + 1 bp subst
Clone 6      atggggaaggctctgacaggagcgggggaaggagacaggagggagggatgggaggggagaagagcccagatggcaggaaa                  mut
Clone 7      atggggaaggctctgacaggagcgggggaaggagacaggagggagggatgggaggggagaagagcccagatggcaggaaa                  mut
Clone 8      atggggaaggctctgacaggagcgggggaaggagacaggagggagggatgggaggggagaagagcccagatggcaggaaa                  mut
Clone 9      atggggaaggctctgacaggagcgggggaaggagacaggagggagggatgggaggggagaagagcccagatggcaggaaa                  mut
Clone 10     atggggaaggctctgacaggagcgggggaaggagacaggaggaagggatgggaggggagaagagcccagatggcaggaaa     mut + 1 bp subst
Clone 11     atggggaaggctctgacaggagcgggggaaggagacaggagggagggatgggaggggagaagagcccagatggcaggaaa                  mut
Clone 12     atggggaaggctctgacaggagcgggggaaggagacaggagggagggatgggaggggagaagagcccagatggcaggaaa                  mut
Clone 13     atggggaaggctctgacaggagcgggggaaggagacaggagggagggatgggaggggagaagagcccagatggcaggaaa                  mut
Clone 14     atggggaaggctctgacaggagcgggggaaggagacaggagggagggatgggaggggagaagagcccagatggcaggaaa                  mut
```

D

Clone 1

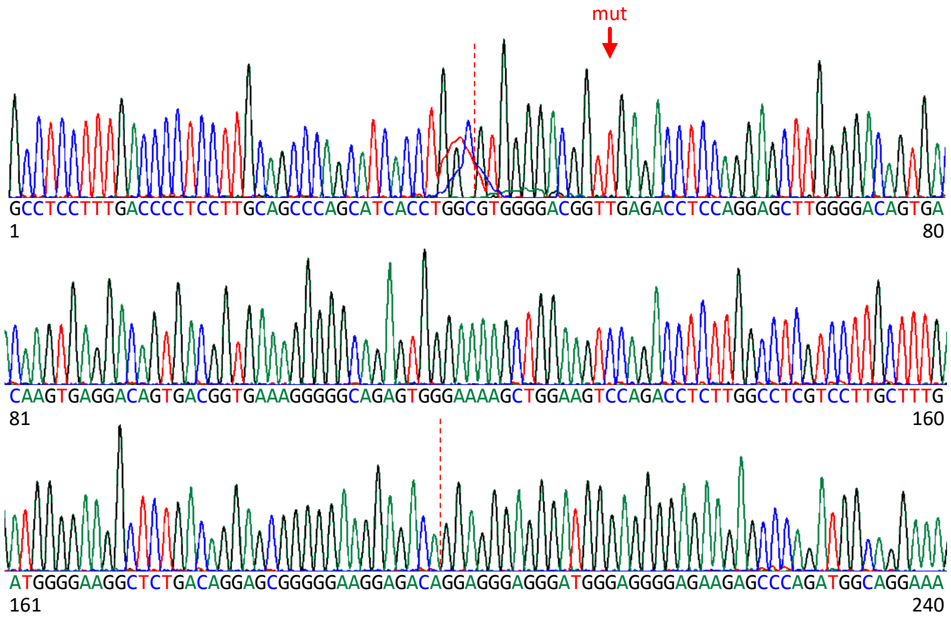

(S8 Fig continued on the next page)

S8 Fig-3

E

	L1CAM exon 14		
	Wild-type	Mutant	
Alexa Fluor 488-positive	100 %	0 %	(n = 14)
Alexa Fluor 488-negative	0 %	100 %	(n = 14)

S8 Fig. Alexa Fluor 488 positivity in the *L1CAM* correction assay represents the reversion of *L1CAM* mut-2 to a wild-type sequence.
The SK-N-BE(2)-derived mut-2 reporter clone was transfected with sgRNA #4 and #6 coupled with Cas9 (H840A) and Donor-*L1CAM* to correct the *L1CAM* mutation via TPN. The L1CAM protein on the surface of transfected cells was labeled with Alexa Fluor 488, and cells were subjected to FCM-based sorting to isolate Alexa Fluor 488-positive and -negative populations. PCR was then performed to amplify a genomic region spanning the mut-2 site within *L1CAM* exon 14 in the Alexa Fluor 488-positive and -negative populations. The amplified PCR products were cloned into a plasmid, and multiple plasmids containing the PCR products as inserts were isolated and sequenced.
(A) DNA sequences of PCR products amplified from Alexa Fluor 488-positive cells. Sequences are shown in alignment with a wild-type control derived from parental SK-N-BE(2) cells displayed at the top. Arbitrary numbers placed above the aligned sequences indicate the relative positions of nucleotides. Blue shading indicates a wild-type sequence resulting from mut-2 reversion. Green letters indicate substituted nucleotides.
(B) Representative sequencing chromatogram obtained in the analysis shown in (A).
(C) DNA sequences of PCR products amplified from Alexa Fluor 488-negative cells displayed in a manner similar to (A). Red shading indicates the mut-2 nonsense mutation.
(D) Representative sequencing chromatogram obtained in the analysis shown in (C).
(E) *L1CAM* genotypes in Alexa Fluor 488-positive and -negative cells determined based on the experimental results shown in (A)–(D). 1-bp substitutions shown in (A) and (C), probably introduced during genome editing or by PCR errors, are not considered in genotyping *L1CAM*, because they are located within an intronic sequence distant from the exon–intron boundary. In (A)–(D), the vertical dotted lines in red indicate a genomic site nicked by Cas9 (H840A) coupled with sgRNA #4 or #6. WT, wild-type; mut, mutant.
