# Supplementary material for: Flow cytometry-based quantification of genome editing efficiency in human cell lines using the L1CAM gene
Source: PLoS One. 2023 Nov 9;18(11):e0294146. doi: 10.1371/journal.pone.0294146 (PMC10635454; doi:10.1371/journal.pone.0294146)
Supplement: S1 Table — (PDF) [file pone.0294146.s009.pdf]

S1 Table. Cas9 target sites with PAMs[a]

Target Name	Sequence
Cas9 nucleases used for L1CAM disruption	
ex1-1	TCCGAGGCCGGCGTGCGCGGAGG
ex2-1	GGTTCAGGCTCCGGCCGGAGGGG
ex2-2	CCTGCTTATCCAGATCCCCGAGG
ex3-1	ttctcagATGAAGGACACCATGg
ex3-2	tgcctggatttctcagATGAAGG
ex4-1	TGAGGCTGATGTCATCTGTGGGG
ex5-1	CCGGATCTCATGGGACATGGCGG
ex26-1	GGGAGCCTCACGCGGCctgaggg
ex26-2	tctcaccctcagGCCGCGTGAGG
ex26-3	TGAGCAGGACGAGGAGCAGGAGG
ex26-4	AGCAAGGGCGGCAAATACTCAGg
ex27-1	GGATAAGGAGGACACCCAGGTGG
ex27-2	GGACACCCAGGTGGACTCTGAGG
ex27-3	ggctcacCTGTACTCGCCGAAGG
ex27-4	tgtgtctccagTGAAGGATAAGG
A Cas9 nuclease used for PIGA disruption	
PIGA-D	ACACAGCTTCCACTGATACCCGG
Cas9 nucleases and nickases used for L1CAM gene correction	
A	CAGGGTGACCTTCACCTGACAGG
B	GGGGTCAAAGGAGGCCTGTCAGG[b]
#1	TTCTTCTCGATTGTGCTGCGGGG
#2	AATCGAGAAGAAAGGTTCCAGGG
#3	CTGCAAGGAGGGGTCAAAGGAGG
#4	GCCCAGCATCACCTGGCGTGGGG
#5	aaagcaaggacgaggccaagagg
#6	agcgggggaaggagacaggaggg
Prime editors used for L1CAM gene correction	
mut-1	GGGGTCAAAGGAGGCCTGTCAGG[b]
mut-2	AGCATCACCTGGCGTGGGGACGG

[a]PAMs, protospacer adjacent motifs

[b]Cas9 nuclease B and the mut-1 prime editor have the same target sequence.

Underlining indicates PAM sequences.

Uppercase and lowercase letters in DNA sequences indicate exonic and intronic sequences, respectively.

Potential off-target sites for the above-listed Cas9 target sites are shown in S2 Table.
