# Supplementary material for: Flow cytometry-based quantification of genome editing efficiency in human cell lines using the L1CAM gene
Source: PLoS One. 2023 Nov 9;18(11):e0294146. doi: 10.1371/journal.pone.0294146 (PMC10635454; doi:10.1371/journal.pone.0294146)
Supplement: S3 Table — (PDF) [file pone.0294146.s011.pdf]

S3 Table. Primer sequences

Primer Name	Sequence
Creation of Donor-L1CAM	
L1CAM intron 11-Fwd-XhoI	CACTCACTCGAGCGATAGCCGAGGGAGATGTAAG
L1CAM exon 17-Rev-XbaI	GCAGTATCTAGAAAGGTGTAGTGGACATAGGGC
PCR after the disruption of L1CAM intron 25–exon 26 boundary	
L1CAM intron 25-Fwd-EcoRI	CACGGCGAATTCCTGCTTCTCCTCCCAGAATC
L1CAM intron 26-Rev-BamHI	GGAGCTGGATCCATCCAGGAGGCCTTGCAGAA
Sequencing of L1CAM intron 25–exon 26 boundary	
pBluescriptII-seq	GCTATGACCATGATTACGCC
PCR after the correction of mut-2 mutation in L1CAM exon 14 (1st PCR)	
L1CAM exon 13-Fwd	ACGAACGCTTCTTCCCCTATG
L1CAM exon 17-Rev	GGGCCATATTTGTTTATGGCAG<br>(locates outside the region homologous to Donor-*L1CAM*)
PCR after the correction of mut-2 mutation in L1CAM exon 14 (2nd PCR)	
L1CAM exon 13-Fwd-BamHI	GCTACTGGATCCTGGCTGCCAATGACCAAA
L1CAM intron 15-Rev-EcoRI	TCTGGAGAATTCGCTTCCACCCTAGGACTTAC
Sequencing of L1CAM exon 14	
L1CAM intron 13-Fwd	TTAAAGGTCAGGCAACCCTTG
RT-PCR and sequencing of L1CAM exon 3	
L1CAM exon 2-Fwd	TGCCCCCACTCCCAACTCCC
L1CAM exon 4-Rev	GCACTTCGGGCTTGCCACTGG

Underlining indicates restriction enzyme recognition sites.
